# *dBRWD3* Regulates Tissue Overgrowth and Ectopic Gene Expression Caused by *Polycomb* Group Mutations

**DOI:** 10.1371/journal.pgen.1006262

**Published:** 2016-09-02

**Authors:** Hsueh-Tzu Shih, Wei-Yu Chen, Kwei-Yan Liu, Zong-Siou Shih, Yi-Jyun Chen, Paul-Chen Hsieh, Kuan-Lin Kuo, Kuo-How Huang, Pang-Hung Hsu, Ya-Wen Liu, Shih-Peng Chan, Hsiu-Hsiang Lee, Yu-Chen Tsai, June-Tai Wu

**Affiliations:** 1 Institute of Molecular Medicine, College of Medicine, National Taiwan University, Taipei, Taiwan; 2 Department of Anatomical Pathology, Far Eastern Memorial Hospital, New Taipei City, Taiwan; 3 Graduate Institute of Toxicology, National Taiwan University College of Medicine, Taipei, Taiwan; 4 Department of Urology, National Taiwan University College of Medicine and Hospital, Taipei, Taiwan; 5 Department of Bioscience and Biotechnology, National Taiwan Ocean University, Keelung, Taiwan; 6 Graduate Institute of Microbiology, College of Medicine, National Taiwan University, Taipei, Taiwan; 7 Genome and Systems Biology Degree Program, College of Life Science, National Taiwan University, Taipei, Taiwan; 8 Department of Life Science and Life Science Center, Tunghai University, Taichung, Taiwan; The University of North Carolina at Chapel Hill, UNITED STATES

## Abstract

To maintain a particular cell fate, a unique set of genes should be expressed while another set is repressed. One way to repress gene expression is through Polycomb group (PcG) proteins that compact chromatin into a silent configuration. In addition to cell fate maintenance, PcG proteins also maintain normal cell physiology, for example cell cycle. In the absence of PcG, ectopic activation of the PcG-repressed genes leads to developmental defects and malignant tumors. Little is known about the molecular nature of ectopic gene expression; especially what differentiates expression of a given gene in the orthotopic tissue (orthotopic expression) and the ectopic expression of the same gene due to *PcG* mutations. Here we present that ectopic gene expression in *PcG* mutant cells specifically requires dBRWD3, a negative regulator of HIRA/Yemanuclein (YEM)-mediated histone variant H3.3 deposition. *dBRWD3* mutations suppress both the ectopic gene expression and aberrant tissue overgrowth in *PcG* mutants through a YEM-dependent mechanism. Our findings identified dBRWD3 as a critical regulator that is uniquely required for ectopic gene expression and aberrant tissue overgrowth caused by PcG mutations.

## Author Summary

Genetic information is stored in our genomic DNA, and different cells retrieve distinct sets of information from our genome. While it is important to activate genomic regions encoding proteins that are essential for a given cell type, it is equally important to silence genomic regions encoding proteins that are potentially harmful to this type of cells. One of the gene silencing mechanisms frequently used during and after development is mediated by the Polycomb group (PcG) proteins. If this guardian function does not perform correctly due to *PcG* mutations, genes that are normally silenced—such as oncogenes—are expressed aberrantly. Due to the activation of oncogenes and the loss of other PcG functions, *PcG* mutant cells often begin to display hallmarks of cancer, such as proliferating beyond control, acquiring stem-cell-like properties, and migrating to distant sites. If the transcriptional mechanisms underlying aberrant gene expression in *PcG-*mutant cancer cells differ from gene expression in normal cells, we may be able to selectively inhibit the growth of cancer cells without affecting their normal counterparts. Here we show that the difference between these two types of gene expression resides in their sensitivity to dBRWD3, a negative regulator of the deposition of histone H3 variant H3.3. Our results indicate that the inactivation of *dBRWD3* or promotion of H3.3 deposition may selectively suppress ectopic gene expression and tumorigenesis driven by mutations in *PcG*.

## Introduction

The eukaryotic genome is packaged in a macromolecular complex termed chromatin. Chromatin is composed of DNA, RNA, histones, and non-histone proteins. The nucleosome, the basic unit of chromatin, consists of a histone octamer containing two copies of histones (H3, H2A, H2B, and H4) and 147 base pairs of DNA wrapped around the octamer [1]. Variants of H2A, H2B, and H3 differ from the canonical histones by a few amino acids [2]. Moreover, canonical histones are encoded by multiple repeated sequence arrays and expressed during the S-phase, while the variants are encoded by single-copy genes and expressed in the interphase [2,3]. Once the histone variants are incorporated into nucleosomes, they confer distinct physical and biochemical properties to DNA templates and thus regulate DNA replication, repair and gene transcription [3,4]. The deposition of histone variants is mediated by specific chaperone complexes. For example, H3.3 deposition, which often occurs in actively transcribed regions, is mediated by a histone chaperone named histone repressor A (HIRA) and its associated chaperone Yemanuclein (YEM) [5–10]. We previously showed that HIRA/YEM activity is negatively regulated by dBRWD3 (Bromodomain and WD repeat-containing protein 3) [11], which adds a second layer of complex regulation to H3.3 deposition. Dendritic arborization of peripheral neurons and photoreceptor development are disrupted in *dBRWD3* mutants. These phenotypes are effectively suppressed by mutations in *yem* or *H3*.*3*, indicating that dBRWD3 functions largely through restricting YEM-dependent H3.3 deposition [11]. However, it remains unknown where in the genome this regulation of H3.3 deposition takes place and how it affects transcription.

Distinct patterns of transcriptional activation and inactivation of the genome contribute to the diversity of cell types in multicellular organisms. To inactivate transcription, *Polycomb* group (PcG) proteins bind to specific genomic regions and modify histones posttranslationally [12,13]. PcG proteins are grouped into two evolutionarily conserved complexes, PRC1 and PRC2. In *Drosophila*, the PRC1 complex consists of Polycomb, Posterior sex combs, Sex combs extra (Sce, the *Drosophila* homolog of human RING1), Polyhomeotic proximal, Polyhomeotic distal with an accessory molecule, and Sex comb on midleg (Scm) [14]. The PRC2 complex is composed of Enhancer of zeste (E(z)), Suppressor of zeste 12, and extra sex combs [13]. Functionally, PRC1 adds a monoubiquitin moiety onto histone H2AK119 (H2AK118 in *Drosophila*), whereas PRC2 catalyzes the trimethylation of H3K27 (H3K27me3). The combined activities of PRC1 and PRC2 repress transcription by compacting chromatin [15]. PcG proteins may also silence gene expression through a compaction-independent mechanism, such as by blocking transcription initiation [16–18].

Misregulation of transcription within typically inactive genomic regions leads to the disorganization of tissues and organisms [19]. For instance, loss of PcG function causes the ectopic expression of *Homeotic* (*Hox*) genes specific to posterior segments and thus disrupts the anterior-to-posterior body plan in embryos [20,21]. In *Drosophila*, loss of PRC1 leads to ectopic expression of Unpaired 1–3, driving aberrant cell proliferation and tissue overgrowth by activating the JAK-STAT pathway [22]. In humans, loss of PRC1 function has been shown to promote tumorigenesis [23,24]. Reduced expression of the PRC1 subunit CBX7 has been implicated in bladder, breast, colon, glioma, lung, pancreatic, and thyroid carcinomas [25–31], and *CBX7* knockout mice develop lung and liver carcinoma [29]. Loss of PRC2 function also causes tumor formation. For example, the tumor-driving H3.3K27M mutation in pediatric diffuse intrinsic pontine gliomas (DIPGs) results in the inactivation of the PRC2 complex, causing ectopic expression of LIN28B, PLAG1, and PLAGL1, and leading to the de-differentiation and hyperproliferation of tumor cells [32–34]. A second mutation in the PRC2 complex genes in patients with neurofibromatosis increases the likelihood of developing malignant peripheral nerve sheath tumors [35,36].

Currently, no therapeutic strategies have been developed for tumorigenesis caused by ectopic gene expression. This is mainly because little is known about how ectopic gene expression is initiated within de-repressed genomic regions, and how it differs from conventional transcription initiation. Here we show that dBRWD3 is specifically required for ectopic gene expression and tissue overgrowth caused by *PcG* mutations. dBRWD3 sustains *PcG* mutation-induced ectopic gene transcription by regulating H3.3 deposition, which in turn affects the way RNA polymerase II occupies transcription start sites. Thus, our results suggest that human BRWD3 could be a potential therapeutic target for PcG mutation-driven tumors.

## Results

### *dBRWD3* mutations suppress ectopic *antennapedia* expression caused by mutations in the PRC1 subunits, *Scm* and *Sce*

In the process of investigating how *dBRWD3* might affect gene expression, we unexpectedly found that the *dBRWD3* mutations suppress the lethality of *Scm* mutants. Similar to other *PcG* mosaic mutants [22], *Scm*^*D1*^ mosaic mutant flies died in the pupal stage. Interestingly, a significant portion of the mosaic *Scm*^*D1*^, *dBRWD3*^*s5349*^ double mutants survived to the adult stage, so did the mosaic *Scm*^*D1*^, *dBRWD3*^*PX2*^ double mutants (Table 1). To explore the relationship between *dBRWD3* and *PcG* genes, we examined the genetic interaction between *dBRWD3* and *Posterior sex comb* (*Psc*), another *PcG* gene. We found that knockdown of *Psc* was semi-lethal, whereas simultaneous knockdown of *Psc* and *dBRWD3* was fully viable (Table 2). Taken together, these results suggest a role for *dBRWD3* as a suppressor of *PcG* genes.

**Table 1:** The genetic interaction between *Scm* and *dBRWD3*.

Genotype	Eclosure rate
*wild type*	95.16% (n = 186)
*Scm*^*D1*^	0% (n = 240)
*Scm*^*D1*^, *dBRWD3*^*s5349*^	11.2% (n = 125)
*Scm*^*D1*^, *dBRWD3*^*PX2*^	10.5% (n = 228)
*dBRWD3*^*s5349*^	9.56% (n = 168)

**Table 2:** The genetic interaction between *Psc* and *dBRWD3*.

Genotype	Eclosure rate
*wild type*	100% (n = 150)
*Psc-dsRNA*	19.2% (n = 166)
*Psc-dsRNA*, *dBRWD3-dsRNA*	100% (n = 136)

**Fig 1:**
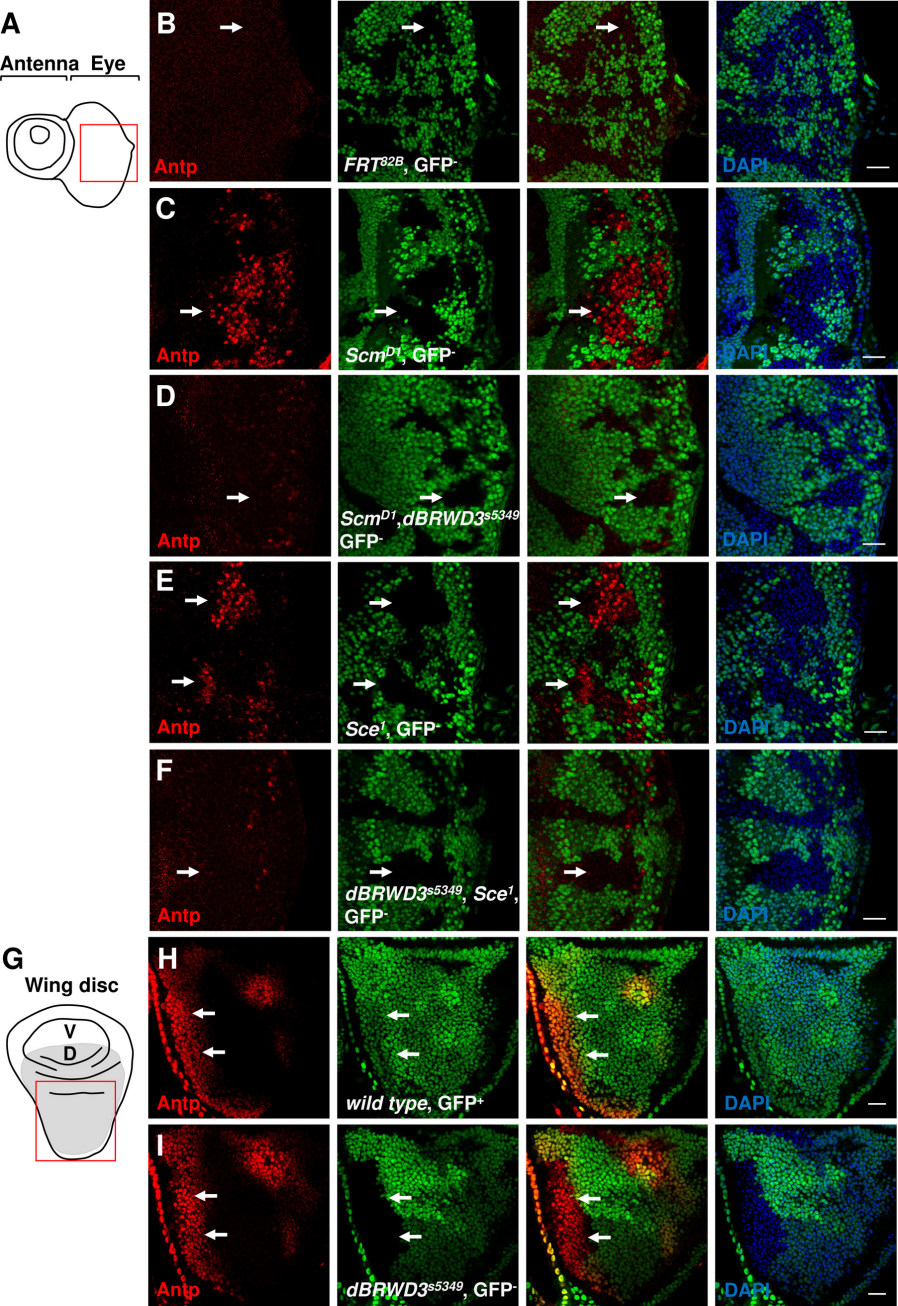
*dBRWD3*^*s5349*^ mutation suppresses ectopic expression of *Antp* in *Scm* ^*D1*^ or *Sce*^*1*^ mutant eye clones. (A) A schematic illustration of a 3^rd^ instar eye imaginal disc. The red square indicates the region examined in the following experiments. (B-F) Antennapedia (Antp) levels (arrows) in *wild-type* clones (B), *Scm*^*D1*^ mutant clones (C), *Scm*^*D1*^, *dBRWD3*^*s5349*^ double-mutant clones (D), *Sce*^*1*^ mutant clones (E), and *dBRWD3*^*s5349*^ *Sce*^*1*^ double-mutant clones (F) generated in the 3^rd^ instar eye imaginal discs by *ey-flp* and marked by the absence of GFP. Scale bars indicate 50μm. (G) A schematic illustration of a 3^rd^ instar wing imaginal disc. V and D stand for ventral and dorsal (marked by grey) compartments, respectively. (H) Antp levels (arrows) in a *wild-type* wing. (I) Antp levels (arrowheads) in *dBRWD3*^*s5349*^ mutant wing disc clones generated by *hs-flp* and marked by the absence of GFP. Scale bars indicate 50μm.

Since ectopic gene expression underlies many phenotypes of *PcG* mutations, we then investigated whether the *dBRWD3* mutations also suppresses ectopic gene expression. While the second thoracic segment-specific *Hox* gene, *antennapedia* (*Antp*), was repressed in *wild-type* eye clones (Fig 1A and 1B, arrow), it was ectopically expressed in *Scm* mutant eye clones located in the posterior region (Fig 1C, arrow). This ectopic *Antp* expression was dramatically reduced in *Scm*^*D1*^, *dBRWD3*^*s5349*^ or *Scm*^*D1*^, *dBRWD3*^*PX2*^ double-mutant eye clones (Figs 1D, S1A, S1B and S1C). Similarly, *Antp* is ectopically expressed in *Sce*^*1*^ mutant eye clones (Fig 1E, arrows) but not in *dBRWD3*^*s5349*^, *Sce*^*1*^ double-mutant clones (Figs 1F and S1D). Interestingly, *dBRWD3* is dispensable for the orthotopic expression of *Antp* in wings (Figs 1G, 1H, 1I and S1E). Overall, these observations reveal that dBRWD3 is involved in the ectopic expression of *Antp* caused by *PcG* mutations.

### Knockdown of *dBRWD3* suppresses ectopic *ultrabithorax* expression in the PRC1 and PRC2 depleted brains

**Fig 2:**
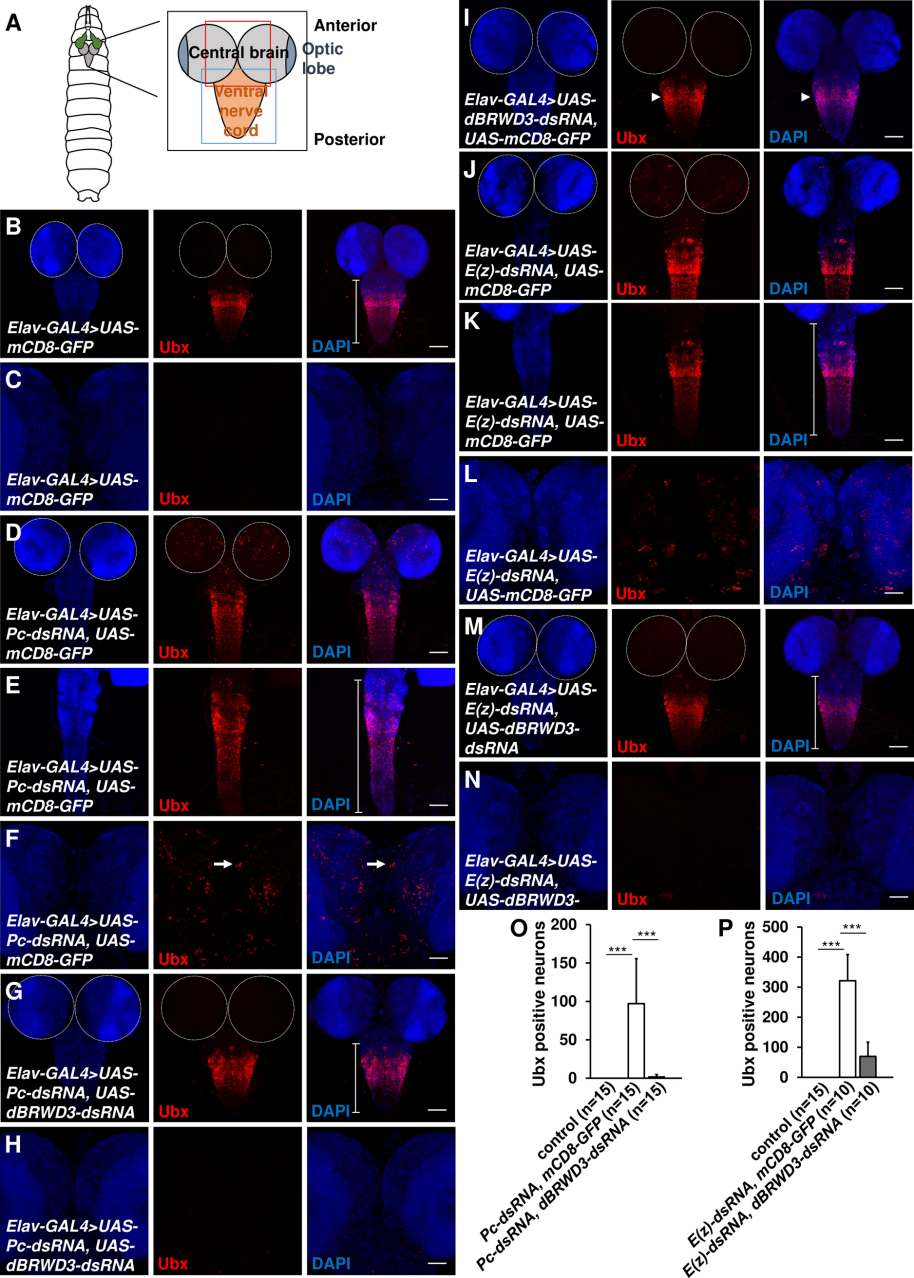
Knockdown of *dBRWD3* suppresses ectopic expression of *Ubx* in *Pc* or *E(z)* depleted brains and condensation failure of *Pc* or *E(z)* depleted ventral nerve cords. (A) A schematic diagram of a 3^rd^ instar central nervous system. The black, red and blue squares indicate the regions examined for the central nervous system, central brain, and ventral nerve cord, respectively. (B and C) Ubx protein levels in the ventral nerve cord (B) and brains (C) of flies expressing *mCD8-GFP* under the control of *Elav-GAL4*. The average number of *Ubx* positive neurons per brain is 0, n = 15. The average length of the ventral nerve cord is 395.6μm, n = 21. (D-F) Ubx protein levels in the ventral nerve cords (D and E) and brains (D and F) of flies expressing *UAS-Pc-dsRNA*, *UAS-mCD8-GFP* under the control of *Elav-GAL4* to deplete *Pc* expression. The average number of *Ubx* positive neurons per brain is 97.1, n = 15, p<0.00001 vs. GAL4 control by Student's t-test. The average length of ventral nerve cord is 468.5μm, n = 49, p<0.001 vs. GAL4 control by Student's t-test. (G and H) Ubx protein levels in the ventral nerve cord (G) and brains (H) of *Pc*, *dBRWD3* doubly depleted flies. The average *Ubx* positive neurons per brain is 1.9, n = 15, p<0.0001 vs. knockdown of *Pc* by Student's t-test. The average length of ventral nerve cords is 389.4μm, n = 45, p<0.00001 vs. knockdown of *Pc* by Student's t-test. (I) Ubx protein (arrowhead) levels in the ventral nerve cord of *dBRWD3* depleted flies. (J-L) Ubx protein levels in the ventral nerve cords (J and K) and brains (J and L) of flies expressing *UAS-E(z)-dsRNA* and *UAS-mCD8-GFP* by *Elav-GAL4*. The average number of *Ubx* positive neurons per brain is 321.6, n = 10, p<0.0001 vs. GAL4 control by Student's t-test. The average length of ventral nerve cords is 456.5μm, n = 39, p<0.01 vs. GAL4 control by Student's t-test. (M and N) Ubx protein levels in the ventral nerve cord (M) and brains (N) of *E(z)*, *dBRWD3* doubly depleted flies. The average number of *Ubx* positive neurons per brain is 69.9, n = 10, p<0.0001 vs. knockdown of *E(z)* by Student's t-test. The average length of the ventral nerve cords is 393.9μm, n = 40, p<0.001 vs. knockdown of *E(z)* by Student's t-test. (O and P) Suppression of *Pc* (O) or *E(z)* (P) depletion-induced ectopic expression of *Antp* by *dBRWD3* depletion. The number of Antp positive neurons in *Pc*, *dBRWD3* (O), *E(z)*, *dBRWD3* (P) doubly depleted brains. *** indicates p<0.0001 by Student's t-test. Brackets indicate the length of the ventral nerve cord. Scale bars indicate 100μm in B, D, G, I, J, and M and 50μm in C, E, F, H, K, L and N.

To determine whether *dBRWD3* suppresses ectopic gene expression other than *Antp* in the eyes, we knocked down *Pc* in the central nervous system by *Elav-GAL4*, reducing the level of *Pc* mRNA to 5% (S2A Fig). *Ubx* is ectopically expressed in the *Pc*-depleted brains but not in the control (Fig 2A, 2B, 2C, 2D, 2F and 2O). In addition, the *Pc* depleted ventral nerve cord was thinner and more elongated compared to the control (Fig 2E, bracket). We found that both ectopic expression of *Ubx* and elongation of ventral nerve cords were suppressed by depletion of *dBRWD3* (Fig 2G and 2H). On the other hand, orthotopic expression of *Ubx* in the ventral nerve cord was not affected in the *dBRWD3*, *Pc* double knockdown (Fig 2G) or in *dBRWD3* knockdown animals (Figs 2I, S2B, S2C and S2D, arrowhead). Thus, ectopic expression of *Ubx* depends on dBRWD3 whereas orthotopic expression of *Ubx* does not.

Depleting *E(z)*, which encodes the H3K27 methyltransferase in PRC2, caused ectopic expression of *Ubx* in brains (Figs 2J, 2L and S2E) and condensation failure in ventral nerve cords (Fig 2K, bracket). The ectopic expression of *Ubx* and condensation failure of ventral nerve cords were also suppressed by knockdown of *dBRWD3* (Fig 2M, 2N and 2P). By contrast, orthotopic expression of *Ubx* was not affected in *dBRWD3*, *E(z)*-doubly depleted ventral nerve cords (S2F, S2G and S2H Fig). Taken together, our data indicates that ectopic *Hox* gene expression depends on dBRWD3 whereas orthotopic *Hox* gene expression does not.

### *dBRWD3*^*s5349*^ suppresses ectopic expression of *unpaired* in *Scm* or *Sce* mutants

**Fig 3:**
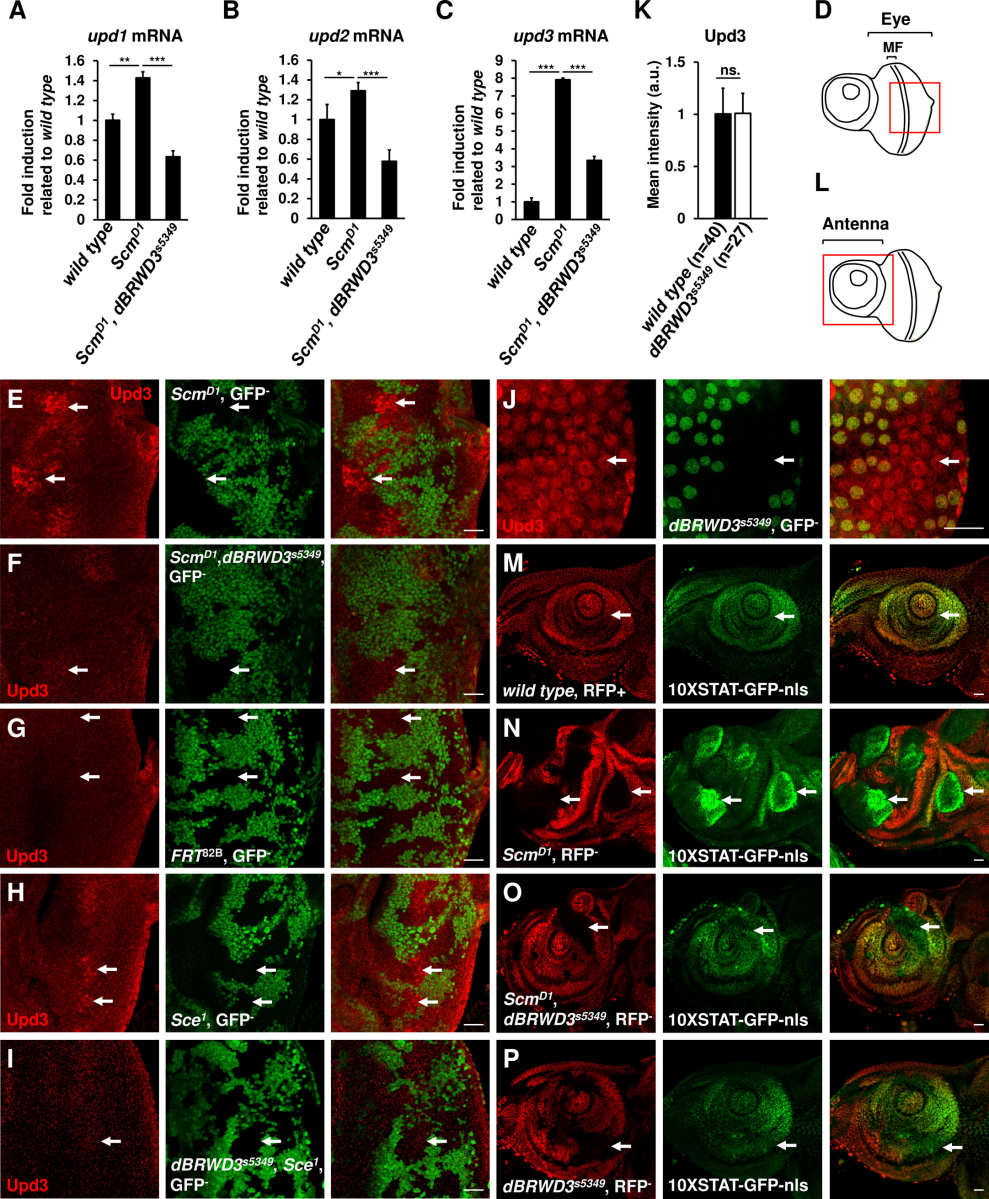
dBRWD3 suppresses ectopic expression of *upd* and activation of the JAK-STAT pathway. (A-C) The *upd1* (A), *upd2* (B), and *upd3* (C) mRNA levels of mosaic eye brain complexes isolated from *wild type*, *Scm*^*D1*^, and *Scm*^*D1*^, *dBRWD3*^*s5349*^ as indicated. Data are shown as means ± S.D. *, **, *** indicate P<0.01, 0.001, 0.0001 respectively by Student's t-test, n = 4. (D) A schematic illustration of a 3^rd^ instar eye imaginal disc. MF stands for morphogenic furrow. The red square indicates the region examined in the following experiments. (E-I) Upd3 levels (arrows) in *Scm*^*D1*^ mutant clones (E), *Scm*^*D1*^, *dBRWD3*^*s5349*^ double-mutant clones (F), *wild-type* clones (G), *Sce*^*1*^ mutant clones (H), and *dBRWD3*^*s5349*^, *Sce*^*1*^ double-mutant clones (I) generated in 3^rd^ instar eye imaginal discs by *ey-flp* and marked by the absence of GFP. Scale bars indicate 50μm. (J) Orthotopic Upd3 levels (arrows) in *dBRWD3*^*s5349*^ mutant clones generated in 2^nd^ instar larval imaginal eye discs marked by the absence of GFP. Scale bars indicate 50μm. (K) Quantification analyses of orthotopic Upd3 levels (arrows) in *dBRWD3*^*s5349*^ mutant clones. ns. indicates not significant. (L) A schematic illustration of a 3^rd^ instar antennal imaginal disc. The red square indicates the region examined in the following experiments. (M) GFP levels (arrows) of the 10XSTAT-nls-GFP reporter in a *wild-type* disc. (N-P) GFP levels (arrows) of the 10XSTAT-nls-GFP reporter in *Scm*^*D1*^ mutant clones (N), *Scm*^*D1*^, *dBRWD3*^*s5349*^ double-mutant clones (O), and *dBRWD3*^*s5349*^ mutant clones (P) generated in 3^rd^ instar antennal imaginal discs by *ey-flp* and marked by the absence of the *ubi* promoter driven mof-RFP. Scale bars indicate 50μm.

In addition to ectopic expression of *Hox* genes, loss of *Ph*, *Psc*, or *Pc* induces ectopic expression of *unpaired (upd) 1–3*, and therefore activation of the JAK-STAT pathway that leads to overgrowth of tumor-like tissues [22,37–39]. By RT-qPCR, we detected mild increases of *upd1* and *upd2* mRNAs (Fig 3A and 3B) and a strong induction of *upd3* mRNA (Fig 3C) in the mosaic *Scm*^*D1*^ mutant brain-eye complex. This upregulation of *upd1-3* was prevented or significantly weakened in the mosaic *Scm*^*D1*^, *dBRWD3*^*s5349*^ double mutants compared with mosaic *Scm*^*D1*^ mutants (Fig 3A, 3B and 3C). Consistently, our immunofluorescent micrographs showed that Upd3 accumulated in *Scm*^*D1*^ mutant clones adjacent to the morphogenetic furrow (Figs 3D, 3E and S3A, arrows), but not in *Scm*^*D1*^, *dBRWD3*^*s5349*^ double-mutant clones (Figs 3F and S3A) or *wild-type* clones (Fig 3G). Similarly, we detected accumulation of Upd3 in *Sce*^*1*^ (Figs 3H and S3B, arrows) and *Sce*^*KO*^ (S4 Fig) mutant clones, but not in the *dBRWD3*^*s5349*^, *Sce*^*1*^ double-mutant clones (Figs 3I and S3B, arrow). On the other hand, we found that orthotopic *upd3* expression in the posterior end of the 2^nd^ instar eye disc was not altered in *dBRWD3*^*s5349*^ mutant clones (Fig 3J and 3K, arrows), indicating that the regulation of *upd3* by dBRWD3 is specific to ectopic expression.

We also used the 10XSTAT-GFP reporter to determine whether the JAK-STAT pathway, which is activated by Upd1-3, is affected by dBRWD3 in PcG mutant cells [40,41]. In contrast to the weak and uniform expression observed in *wild-type* antennal discs (Fig 3L and 3M, arrows), the GFP signal was much higher in *Scm*^*D1*^ mutant clones (Fig 3N), likely due to up-regulation of *upd1-3*. It remained unchanged in *Scm*^*D1*^, *dBRWD3*^*s5349*^ double mutants (Fig 3O, arrow). The STAT activity in the antennal disc was not affected in *dBRWD3*^*s5349*^ single mutants (Fig 3P, arrow). Given these results, we propose that *dBRWD3*^*s5349*^ suppresses ectopic activation of the JAK-STAT pathway caused by *Scm*^*D1*^ or *Sce*^*1*^ mutations.

### *dBRWD3*^*s5349*^ suppresses tissue overgrowth caused by the loss of PcG function

**Fig 4:**
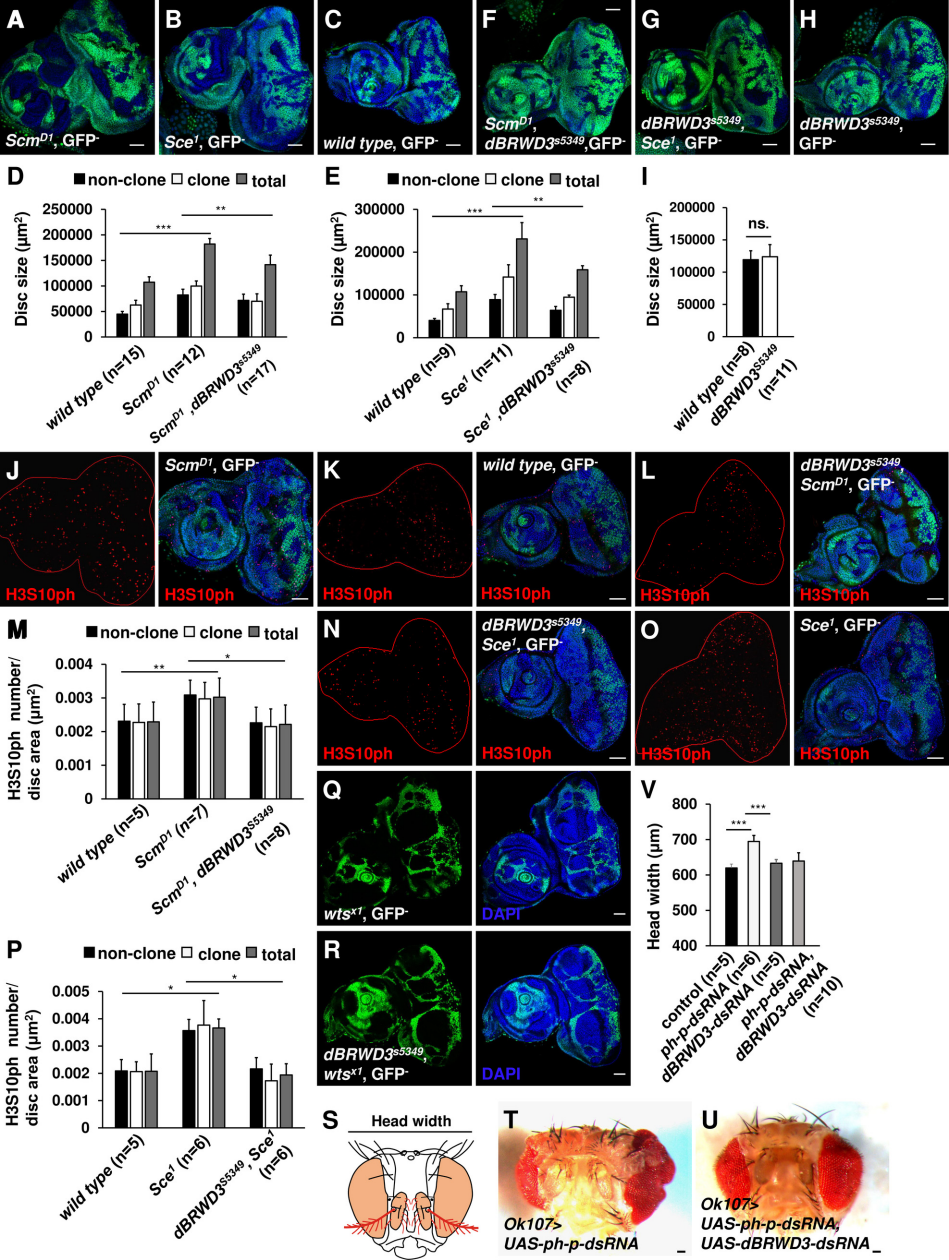
*dBRWD3*^*s5349*^ suppresses tissue overgrowth caused by *PcG* mutations. (A-C) *Scm*^*D1*^(A)_,_ *Sce*^*1*^(B), and *wild-type* (C) mosaic clones were generated in 3^rd^ instar eye antennal discs by *ey-flp* and marked by the absence of GFP. Scale bars indicate 50μm. (D and E) Bar chart presentations of clonal, non-clonal, and total eye antennal disc area of wild type, *Scm*^*D1*^, and *Scm*^*D1*^, *dBRWD3*^*s5349*^ (D), and wild type, *Sce*^*1*^, and *dBRWD3*^*s5349*^, *Sce*^*1*^ (E). Data are shown as means ± S.D. **, *** indicate P<0.001, 0.0001 respectively by Student's t-test. ns. indicates not significant. (F-H) *Scm*^*D1*^, *dBRWD3*^*s5349*^ (F), *dBRWD3*^*s5349*^, *Sce*^*1*^ (G), or *dBRWD3*^*s5349*^ (H) mosaic clones were generated in 3^rd^ instar eye antennal discs by *ey-flp* and marked by the absence of GFP. Scale bars indicate 50μm. (I) Bar chart presentations of clonal, non-clonal, and total eye antennal disc areas of wild type and *dBRWD3*^*s5349*^. Data are shown as means ± S.D. ns. indicates not significant by Student's t-test. (J-L) H3S10ph levels (red) in *Scm*^*D1*^ (J), *wild type* (K), *Scm*^*D1*^, *dBRWD3*^*s5349*^ (L), mosaic mutant eye discs. Scale bars indicate 50μm. (M) The clonal, non-clonal, and total density of H3S10ph positive cells of wild type, *Scm*^*D1*^, and *Scm*^*D1*^, *dBRWD3*^*s5349*^ mosaic eye antennal discs. Data are shown as means ± S.D. *, ** indicate p<0.01, 0.001 respectively by Student's t-test. (N and O) H3S10ph levels (red) in *dBRWD3*^*s5349*^, *Sce*^*1*^ (N), and *Sce*^*1*^ (O) mosaic mutant eye discs. Scale bars indicate 50μm. (P) The clonal, non-clonal, and total density of H3S10ph positive cells of wild type, *Sce*^*1*^, and *dBRWD3*^*s5349*^, *Sce*^*1*^ mosaic eye antennal discs. Data are shown as means ± S.D. * indicates p<0.01 by Student's t-test. (Q and R) *wts*^*x1*^ (Q), *dBRWD3*^*s5349*^, *wts*^*x1*^ (R) mosaic clones were generated in 3^rd^ instar eye antennal discs by *ey-flp* and marked by the absence of GFP. Scale bars indicate 50μm. (S) A schematic illustration of an adult head. Colored areas indicate *OK107-GAL4* expression domains. (T) Eye phenotype produced by knockdown of *polyhomeotic proximal* (*ph-p*) by the *OK-107 GAL4* driver. The scale bar indicates 50μm. (U) An adult eye that resulted from double knockdown of *ph-p* and *dBRWD3*. Scale bars indicate 50μm. (V) Head width of *ph-p*-depleted, *dBRWD3*-depleted, and *dBRWD3*, *ph-p*-doubly-depleted eyes. *** indicates p<0.0001 by Student's t-test.

By ectopically expressing Upd1, Upd2, and Upd3, mutations in PRC1 and PRC2 complexes cause cell autonomous and non-autonomous proliferations [22,37]. Consistently, we found that *Scm*^*D1*^ (Fig 4A) or *Sce*^*1*^ (Fig 4B) mutant clones were larger than *wild-type* clones (Fig 4C, 4D and 4E). Since Upd3 is a diffusible ligand stimulating non-cell-autonomous proliferation, we found the non-clonal area of mosaic *Scm*^*D1*^ (Fig 4D) or *Sce*^*1*^ (Fig 4E) eye antennal discs were also larger. Overall, mosaic *Scm*^*D1*^ or *Sce*^*1*^ mutant discs were 1.5- (Fig 4D) or 1.8-fold in size (Fig 4E) compared to *wild-type* respectively. This tissue overgrowth could be suppressed by *dBRWD3*^*s5349*^ (Fig 4D, 4E, 4F and 4G). As a control, the mosaic *dBRWD3*^*s5349*^ alone did not reduce the disc size (Fig 4H and 4I).

Quantitatively, the numbers of clonal, non-clonal, and total mitotic cells marked by phosphorylation of H3S10 (H3S10ph) were increased in mosaic *Scm*^*D1*^ mutant eye-antennal disc (Fig 4J). It was reduced to a *wild-type* level in mosaic *Scm*^*D1*^, *dBRWD3*^*s5349*^ mutant eye-antennal discs (Fig 4K, 4L and 4M). A similar suppression of proliferation was found in mosaic *dBRWD3*^*s5349*^, *Sce*^*1*^ mutant eye-antennal discs (Fig 4N) as opposes to *Sce*^*1*^ mutants (Fig 4O and 4P). The elongated *Pc* and *E(z)* depleted ventral nerve cords and control ventral nerve cord had comparable mitotic indices and undetectable expression of *upd1*, *upd2* and *upd3*, indicating that the elongation of the ventral nerve cord was not caused by excessive proliferation.

To determine whether the *dBRWD3* mutation also suppresses other types of oncogenic tissue overgrowth, we sampled tissue overgrowth caused by the *warts* (*wts*) mutation that activates the hippo pathway [42,43]. We found that *dBRWD3*^*s5349*^ did not suppress the expression of the hippo pathway target gene, *expanded* (*ex*) (S5A and S5B Fig) and tissue overgrowth (Fig 4Q and 4R). Thus, the *dBRWD3* mutation appears to suppress the oncogenic tissue overgrowth specifically related to *PcG* mutations. To examine the growth-inhibition effect of the *dBRWD3* mutation beyond the developmental stage, we generated overgrown eyes and surrounding tissues by knockdown of *ph-p* (Fig 4S and 4T). In *dBRWD3* and *ph-p* double-knockdown eyes, the tissue overgrowth phenotype was suppressed (Fig 4U and 4V). From these data, we infer that the tissue overgrowth induced by *PcG* gene depletion requires *dBRWD3*.

### H3.3 accumulation correlates with suppression of ectopic gene expression mediated by loss of *dBRWD3*

**Fig 5:**
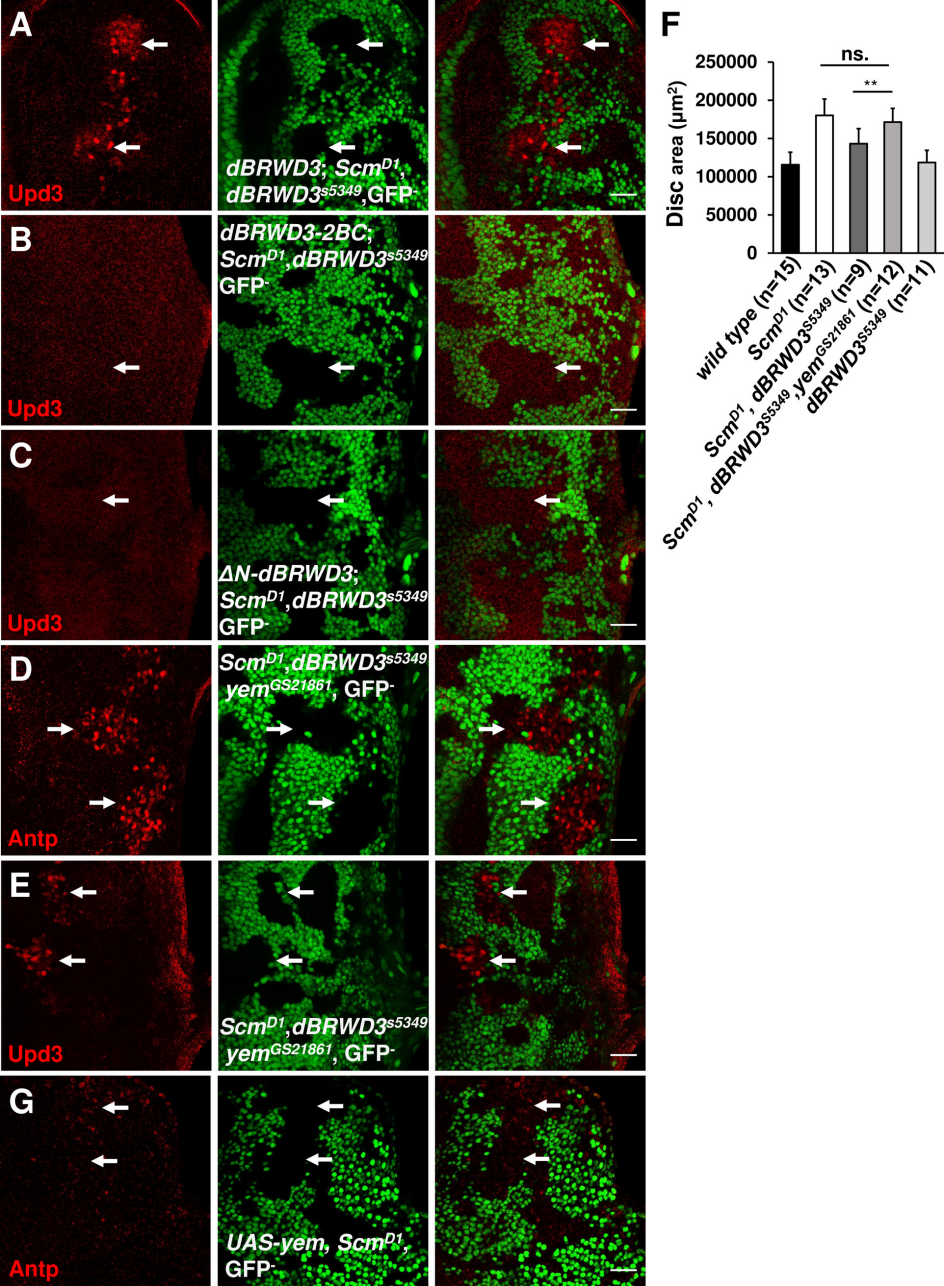
dBRWD3 regulates ectopic gene expression and clone size in a YEM-dependent manner. (A-C) upd3 levels (arrows) in *Scm*^*D1*^, *dBRWD3*^*s5349*^ double-mutant clones complemented with wild type *dBRWD3-RFP* (A), *dBRWD3-2BC-RFP* (B) or *ΔN-dBRWD3-RFP* (C). Scale bars indicate 50μm. (D and E) Antp (D) and upd3 (E) levels (arrows) in *Scm*^*D1*^, *dBRWD3*^*s5349*^, *yem*^*GS21861*^ mosaic mutant clones in eye discs. Scale bars indicate 50μm. (F) Disc sizes of mosaic *Scm*^*D1*^, *Scm*^*D1*^ *dBRWD3*^*s5349*^ double-mutant, and *Scm*^*D1*^, *dBRWD3*^*s5349*^, *yem*^*GS21861*^ triple-mutant eye antennal discs. Data are shown as means ± S.D. ** indicates p<0.001 by Student's t-test. ns. indicates not significant. (G) Antp protein levels in mosaic *Scm*^*D1*^ mutant eye antennal discs expressing *yem-Flag* under the control of *GMR-GAL4*. Scale bars indicate 50μm.

dBRWD3 contains bromodomain I and II (BRDI and BRDII) that were predicted to be acetylated histone-binding domains (S6A Fig) [44]. To investigate the function of these bromodomains, we complemented *Scm*^*D1*^, *dBRWD3*^*s5349*^ double-mutant cells with wild-typ*e dBRWD3*, *dBRWD3-N1287A*, and *dBRWD3-N1451A*, in which the conserved asparagines in the BC loops of BRDI and BRDII were mutated into alanines. The wild-type *dBRWD3-RFP*, *dBRWD3-N1287A-RFP*, and *dBRWD3-N1451A-RFP* could restore the ectopic *upd3* expression in *Scm*^*D1*^, *dBRWD3*^*s5349*^ double-mutant cells (Figs 5A, S6B and S6C). However, when both the BRDI and BRDII were disrupted, the *dBRWD3-N1287A*, *N1451A-RFP* (designated as the *dBRWD3-2BC-RFP*) could not restore the ectopic expression of *upd3* (Fig 5B). This failure to complement is not related to expression levels because *dBRWD3-2BC-RFP* was expressed more than wild-type *dBRWD3-RFP* (S7 Fig). Therefore, the BRDI and BRDII of dBRWD3 are functionally redundant in supporting ectopic gene expression. We also complemented *Scm*^*D1*^, *dBRWD3*^*s5349*^ double-mutant cells with a HLH motif-deleted, *ΔN-dBRWD3*, which no longer interacts with the DNA damage binding protein 1 (DDB1) and cannot be recruited to cullin4/DDB1 organized E3 ligase [11]. The *ΔN-dBRWD3-RFP* also failed to restore the ectopic *upd3* expression in *Scm*^*D1*^, *dBRWD3*^*s5349*^ double-mutant cells (Fig 5C). Together, these results suggest that the activities of dBRWD3 binding to acetylated histones and cullin 4/DDB1 organized E3 ligase are essential for maintaining ectopic gene expression.

Previously, we demonstrated that dBRWD3 limits HIRA/YEM-mediated H3.3 deposition [11]. However, it is not clear whether the BRDI, BRDII, and HLH motif of dBRWD3 are important for dBRWD3 regulation of H3.3. When we complemented *dBRWD3*^*s5349*^ mutant cells with *ΔN-dBRWD3-RFP* or *dBRWD3-2BC-RFP*, the H3.3 levels in the *dBRWD3*^*s5349*^ mutant cells remained higher than those in *wild-type* cells (S8A and S8B Fig, arrows). By contrast, *dBRWD3-N1287A* and *dBRWD3-N1451A* reduced the H3.3-dendra2 to a normal level (S8C and S8D Fig, arrows), indicating a negative correlation between accumulation of H3.3 and ectopic gene expression. Indeed, the negative correlation was also observed in the *dBRWD3* knockdown brains, where the endogenous H3.3 levels were higher than in control brains (S8E Fig). When we co-immuno-stained the mosaic discs with ant-Antp antibody, the ectopic anti-Antp signals were strongly reduced along with accumulated H3.3 in *Scm*^*D1*^, *dBRWD3*^*s5349*^ double-mutant clones (S9B Fig, arrows).

### *dBRWD3*^*s5349*^ suppresses *Scm*^*D1*^ through a *yemanuclein* (*yem*)-dependent mechanism

We next examined whether the increased H3.3 deposition suppresses ectopic gene expression. To this end, we introduced a *yem* mutation to reduce the *dBRWD3* mutation-induced H3.3 deposition (S9C Fig) [11]. In *Scm*^*D1*^, *dBRWD3*^*s5349*^, *yem*^*GS21861*^ triple-mutant clones, the ectopic expression of *Antp* was restored and coincided with the reduction of H3.3 (Figs 5D, S9C and S10A), suggesting that the *dBRWD3* mutation suppresses the *Scm* mutation through a YEM-dependent mechanism. Moreover, *upd3* was ectopically expressed in this triple mutant clone (Figs 5E and S10B). Consistently, the size of the *Scm*^*D1*^, *dBRWD3*^*s5349*^, *yem*^*GS21861*^ triple-mutant eye-antennal disc was larger than that in the *Scm*^*D1*^, *dBRWD3*^*s5349*^ double-mutant (Fig 5F). To further substantiate the role for H3.3 in ectopic gene expression, we investigated whether ectopic gene expression could be suppressed by YEM-induced H3.3 deposition (S11A, S11B and S11C Fig) without any mutation in *dBRWD3*. YEM over-expression effectively suppressed the ectopic expression of *Antp* (Figs 5G and S10C). Taken together, these data indicate that dBRWD3 supports ectopic gene expression and tissue overgrowth mediated by *PcG* mutations by limiting HIRA/YEM-mediated H3.3 deposition.

### *dBRWD3* is epistatic to *trithorax* during ectopic gene expression through its role in maintaining PolII at the proximal region

**Fig 6:**
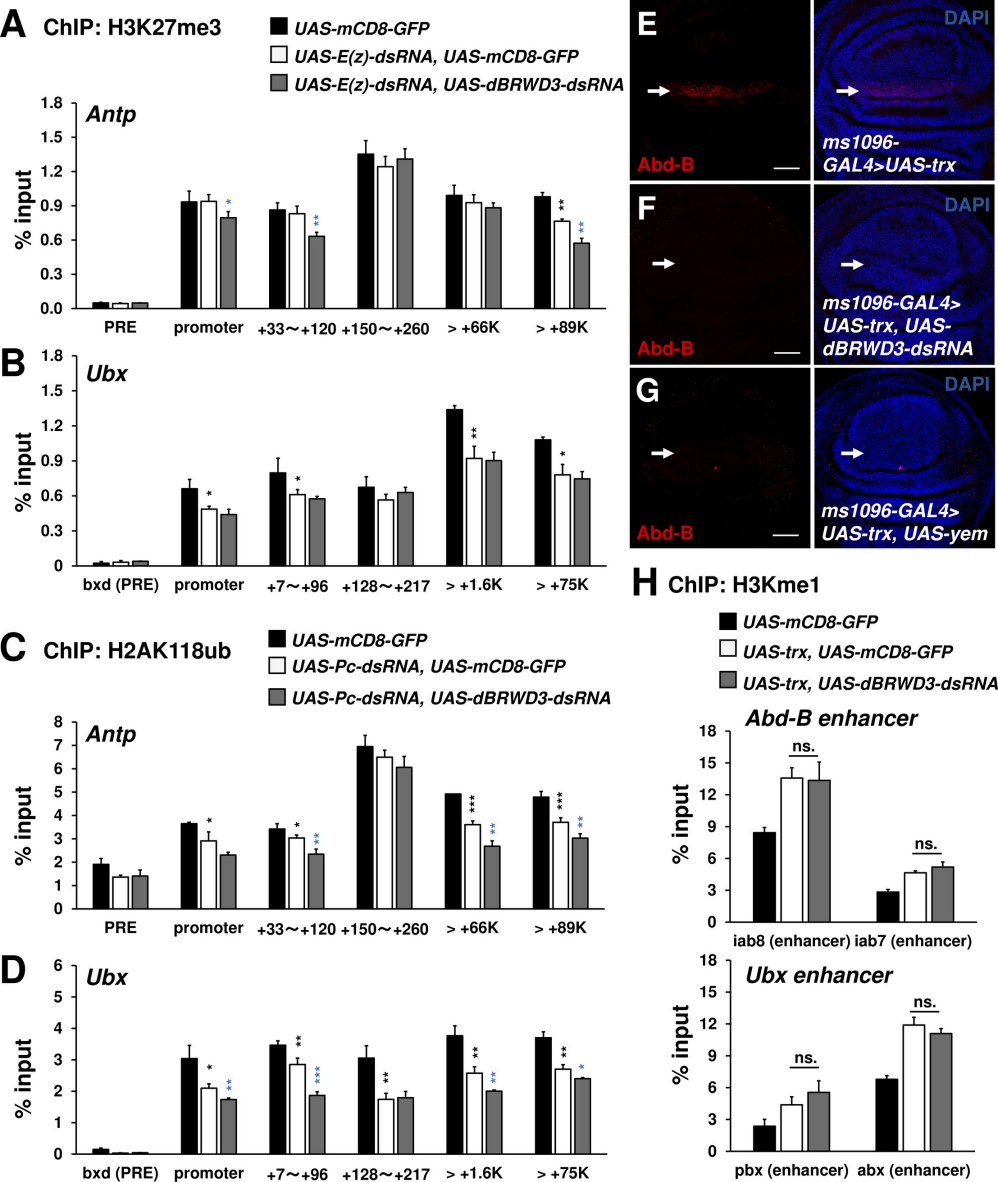
*dBRWD3* is epistatic to *trx* in the ectopic expression of *Ubx* and *Abd-B*. (A and B) A ChIP-qPCR analysis of H3K27me3 levels at the enhancers, promoters, and transcription start sites of *Antp* (A) and *Ubx* (B) in *Elav-GAL4* control, *E(z)* depleted, and *E(z)*, *dBRWD3* doubly depleted brains. Black asterisks indicate control versus *E(z)* depletion. Blue asterisks indicate *E(z)* depletion versus *E(z)*, *dBRWD3* double depletion. (C and D) A ChIP-qPCR analysis of H2AK118ub levels at the enhancers, promoters, and transcription start sites of *Antp* (C) and *Ubx* (D) in *Elav-GAL4* control, *Pc* depleted, and *Pc*, *dBRWD3* doubly depleted brains. Black asterisks indicate control versus *Pc* depletion. Blue asterisks indicates *Pc* depletion versus *Pc*, *dBRWD3* double depletion. (E-G) *trx* was overexpressed under the control of *ms-1096-GAL4*. The TRX-induced Abd-B expression (arrows) in *wild-type* (E), *dBRWD3* depletion (F), and *yem* over-expression (G) backgrounds. Scale bars indicate 20μm. (H) A ChIP-qPCR analysis of H3K4me1 levels at *Ubx* and *Abd-B* enhancers in the *UAS-mCD8-GFP* control, *trx* over-expression, and *trx* over-expression, *dBRWD3* depleted wings as indicated. ns. indicates not significant. ChIP-qPCR Data are shown as means ± S.D from 4 technical replicates. *, **, *** indicate P<0.05, 0.01, 0.001 respectively by Student's t-test.

To understand how the *dBRWD3* mutation suppresses ectopic gene expression, we investigated whether dBRWD3 is required for the removal of pre-existing H3K27me3 and H2A118ub at *Antp* and *Ubx* loci upon depletion of *E(z)* and *Pc*. The ChIP-qPCR analysis revealed a reduction of H3K27me3 in the distal region of *Antp* and at *Ubx* when *E(z)* was depleted (Fig 6A and 6B). Similarly, H2A118ub levels at *Antp* and *Ubx* were also decreased in the *Pc* depleted brains (Fig 6C and 6D). When *dBRWD3* was depleted by RNAi together with *E(z)* or *Pc*, the H3K27me3 or H2A118ub levels at *Antp* and *Ubx* loci remained low or became lower (Fig 6A, 6B, 6C and 6D), indicating that knockdown of *dBRWD3* promotes or does not affect the removal of the pre-existing H3K27me3 and H2A118ub. Moreover, dBRWD3 is not required for the removal of pre-existing H3K27me3 in the *E(z)* depleted wings at the global level, as revealed by the equally reduced H3K27me3 immunostaining signals in the *E(z)* knockdown, and *E(z)*, *dBRWD3* double-knockdown wings (S12A, S12B and S12C Fig). H3K27me3 levels were not changed in the *Pc* knockdown, and *Pc*, *dBRWD3* double-knockdown wings compared with the control (S12D, S12E, S12F Fig). Similarly, *dBRWD3* was not required for the removal of pre-existing H2AK118ub in the *Sce* mutant clones at the global level (S12G and S12H Fig). *trithorax* (*trx*) encodes an H3K4 monomethyltransferase [45] and antagonizes PcG activity by binding to PRE sites, the enhancer cis-elements targeted by PcG proteins. In different cellular contexts, ectopic gene expression might or might not depend on *trx* [46,47]. To investigate the requirement of *trx* in ectopic expression of *Antp* in eyes, we generated *Scm*^*D1*^, *trx*^*E2*^ double-mutant eye clones and found that the ectopic expression of *Antp* was suppressed (S13 Fig), indicating that *trx*, like *dBRWD3*, is required for ectopic *Antp* expression. We next investigated whether *dBRWD3* and *trx* function in a linear pathway or in parallel. We found that over-expression of *trx* in the eye disc proper, peripodial epithelium of the eye disc, and wing disc was sufficient to induce ectopic expression of *Antp* or *Abd-B* (Figs 6E, S14A, S14B and S14D), but not in a *dBRWD3* knockdown background (Figs 6F, S14C and S14E). In addition, Trx-induced *Ubx* ectopic expression in wing discs was strongly suppressed by knockdown of *dBRWD3*, albeit incompletely (S14F and S14G Fig). It seems that H3.3 deposition underlies the suppression of this ectopic gene expression, since Trx-induced *Abd-B* ectopic expression was also completely suppressed by YEM over-expression (Fig 6G).

**Fig 7:**
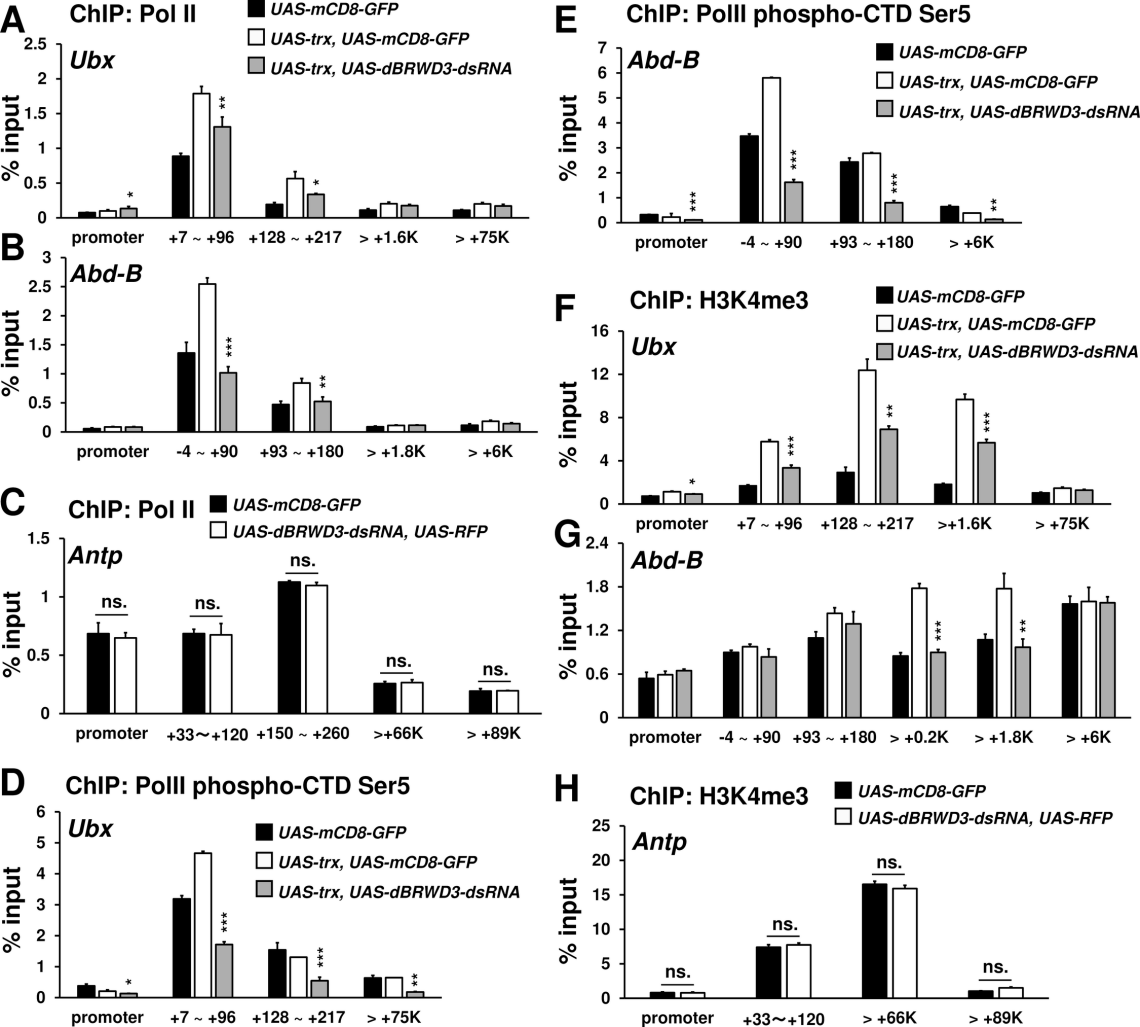
*dBRWD3* is required for maintaining PolII and H3K4me3 levels at the 5' end of *Ubx* and *Abd-B*. (A and B) A representative ChIP-qPCR analysis of PolII levels at *Ubx* (A) and *Abd-B* (B) in the *UAS-mCD8-GFP* control, in wings over-expressing *trx*, or in wings with concurrent *trx* over-expression and *dBRWD3* depletion. Black asterisks indicate *trx* over-expression versus *trx* over-expression and *dBRWD3* depletion. (C) A representative ChIP-qPCR analysis of PolII levels at the promoters and transcription start sites of *Antp* in the *UAS-mCD8-GFP* control and *dBRWD3*-depleted wings. ns. indicates not significant. (D and E) Similar to (A and B), a ChIP-qPCR analysis of PolII phospho-CTD Ser5 levels at *Ubx* (D) and *Abd-B* (E). Black asterisks indicate *trx* over-expression versus *trx* over-expression and *dBRWD3* depletion. (F and G) Similar to (A and B), a representative ChIP-qPCR analysis of H3K4me3 levels at *Ubx* (F) and *Abd-B* (G). Black asterisks indicate *trx* over-expression versus *trx* over-expression and *dBRWD3* depletion. (H) Similar to (C), a representative ChIP-qPCR analysis of H3K4me3 levels at the promoters and transcription start sites of *Antp* in the *UAS-mCD8-GFP* control and *dBRWD3*-depleted wings. ns. indicates not significant. ChIP-qPCR Data are shown as means ± S.D from 4 technical replicates. *, **, *** indicate P<0.05, 0.01, 0.001 respectively by Student's t-test.

To understand how dBRWD3 affects Trx-induced ectopic gene expression, we used ChIP to determine the levels of H3K4me1 and PolII over *Ubx* and *Abd-B* loci. Compared to the control, Trx increased H3K4me1 levels at the enhancer regions of *Ubx* and *Abd-B* irrespective of *dBRWD3* depletion (Fig 6H), which was later found to have no effects on Trx-induced monomethylation of H3K4 on a global scale (S15A, S15B, S15C, S15D, and S15E Fig). These data indicate that *dBRWD3* is epistatic to *trx* with respect to ectopic gene expression. By contrast, Trx-induced PolII levels at the transcription start sites and 5' ends of *Ubx* and *Abd-B* were significantly reduced when *dBRWD3* was depleted by RNAi (Fig 7A and 7B), while the PolII levels for orthotopically expressing *Antp* were not affected (Fig 7C). When *trx* is overexpressed in the wing imaginal discs, RNA PolII increased on the *Antp* promoter, which is likely contributed by the purely orthotopic *Antp* expression and the Trx-induced ectopic *Antp* expression. The additional knockdown of *dBRWD3* restored the PolII occupancy to a level similar to the orthotopic *Antp* expression control (S16 Fig), indicating that knockdown of *dBRWD3* suppressed only the Trx-induced increase of PolII occupancy but not the PolII occupancy of orthotopically expressing *Antp*. Similarly, PolII phospho-CTD Ser5 levels around the transcription start sites of *Ubx* and *Abd-B* were reduced upon knockdown of *dBRWD3* (Fig 7D and 7E). We also detected higher levels of H3K4me3, a marker for active chromatins, at the transcription start sites and 5' ends of *Ubx* and *Abd-B* upon *trx* over-expression in a *dBRWD3*-dependent manner (Fig 7F and 7G). Nevertheless, the levels of H3K4me3 at the orthotopically expressed *Antp* were not sensitive to *dBRWD3* depletion (Fig 7H). These observations suggest that, in ectopic gene expression, dBRWD3 is required for the activation of chromatin specifically at transcription start sites but not in enhancer regions.

**Fig 8:**
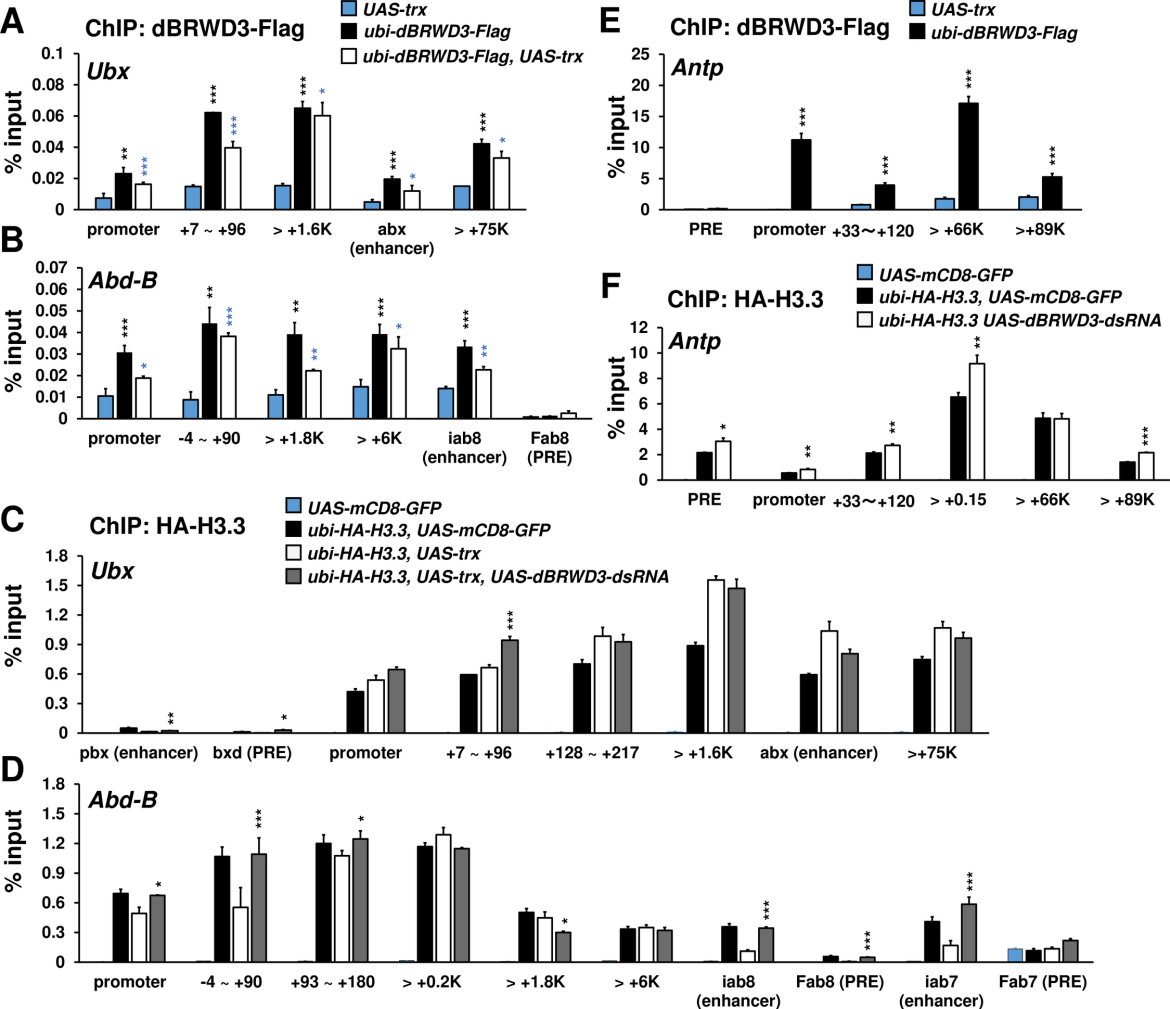
dBRWD3 negatively regulates H3.3 at the promoter and 5' regions of *Ubx* and *Abd-B* in the *trx* over-expressing wing discs. (A and B) A ChIP-qPCR analysis of dBRWD3-Flag levels at *Ubx* (A) and *Abd-B* (B). Black asterisks indicate negative control versus *dBRWD3-Flag*. Blue asterisks indicate negative control versus *dBRWD3-Flag* and *trx* over-expression. (C and D) A representative ChIP-qPCR analysis of HA-H3.3 levels at *Ubx* (C) and *Abd-B* (D) in the *UAS-mCD8-GFP* control, the *trx* over-expressing, and the *trx* over-expressing, *dBRWD3* depleted wings as indicated. Black asterisks indicate *trx* over-expression versus *trx* over-expression and *dBRWD3* depletion. (E) A ChIP-qPCR analysis of dBRWD3-Flag levels at *Antp*. Black asterisks indicate negative control versus *dBRWD3-Flag*. (F) A representative ChIP-qPCR analysis of HA-H3.3 levels at *Antp*. Black asterisks indicate control versus *dBRWD3* depletion. ChIP-qPCR Data are shown as means ± S.D from 4 technical replicates. *, **, *** indicate P<0.05, 0.01, 0.001 respectively by Student's t-test.

To examine whether the reduction of PolII and H3K4me3 are directly caused by H3.3 deposition, we examined dBRWD3 and H3.3 levels across *Ubx* and *Abd-B* loci. ChIP revealed that dBRWD3 was present at these regions and Trx over-expression moderately reduced the occupancy of dBRWD3 (Fig 8A and 8B). Although dBRWD3 was recruited to the promoters, 5' and 3' regions of these ectopically expressed loci, it predominantly reduced H3.3 levels at the transcription start sites (Fig 8C and 8D). These results imply that dBRWD3 maintains PolII levels of ectopically expressed genes by limiting H3.3 deposition at the transcription start sites. Moreover, dBRWD3 was also present at *Antp* locus (Fig 8E) and limited H3.3 levels at the promoters and the transcription start site of *Antp* (Fig 8F). Nevertheless, PolII and H3K4me3 levels at *Antp* were not affected by knockdown of *dBRWD3* (Fig 7C and 7H), indicating that the sensitivity toward H3.3 but not H3.3 levels at the promoter and transcription start site *per se* distinguishes ectopic gene expression from orthotopic gene expression.

### Loss of TFII-D or TFII-H activities suppresses ectopic gene expression

**Fig 9:**
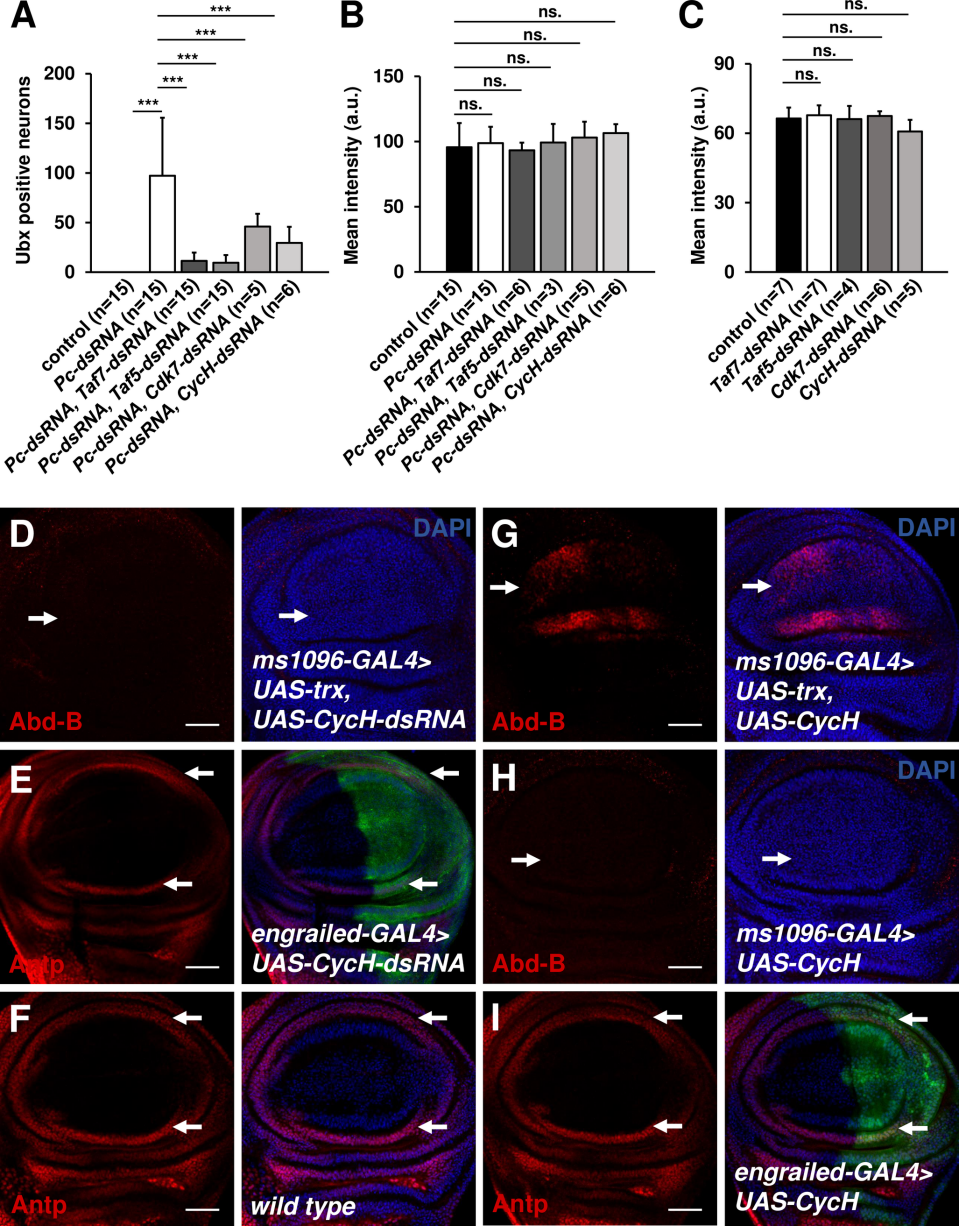
Ectopic expression of *Antp* is more sensitive to partial knockdown of *Taf5*, *Taf7*, *Cdk7*, and *CycH*. (A) Suppression of *Pc* depletion-induced ectopic expression of *Ubx* by depletion of *Taf5*, *Taf7*, *Cdk7*, and *CycH*. Quantification of Ubx positive neuron number in the doubly depleted brains as indicated. (B) The orthotopic expression of *Ubx* in the doubly depleted ventral nerve cords as indicated. (C) The orthotopic expression of *Ubx* in control, *Taf5*, *Taf7*, *Cdk7*, and *CycH* depleted ventral nerve cords. *** indicates p<0.0001 by Student's t-test. ns. indicates not significant. (D) The Trx-induced Abd-B expression (arrows) in the *CycH* knockdown wings. (E and F) The orthotopic expression of *Antp* in the *CycH* knockdown (E), and the control (F) wings. (G and H) The Abd-B expression in wings over-expressing both *trx* and *CycH* (G) or *CycH* alone (H) under the control of *ms1096-GAL4*. (I) The orthotopic expression of *Antp* in the *CycH* over-expression wings. Scale bars indicate 20μm.

Since in ectopic gene expression PolII occupancy appears to be more sensitive to H3.3 deposition at transcription start sites, we speculated that the initiation of ectopic transcription is more vulnerable to perturbation. To test this hypothesis, we reduced the activities of the general transcriptional factor TFII-D by knocking down various TATA box-binding protein (TBP)-associated factors (TAF). Although TAFs are essential factors, animals with 65% reduction of *Taf5* or 40% reduction of *Taf7* in the central nervous system can grow to the adult stage without discernible defects (S17A and S17B Fig). *Ubx*-expressing neurons in *Pc*, *Taf5* or *Pc*, *Taf7* double-knockdown brains exhibited 88% or 90% reduction in number relative to *Pc* depleted brains, respectively (Fig 9A). Next, we investigated whether ectopic expression is more sensitive to general transcriptional factor TFII-H subunits, Cdk7 and CycH, which phosphorylate PolII CTD serine 5. Similarly, *Pc*, *Cdk7* or *Pc*, *CycH* double depletion by RNAi significantly reduced the number of neurons ectopically expressing *Ubx* in brains (Figs 9A, S17C and S17D). By contrast, orthotopic expression of *Ubx* in ventral nerve cords was not affected by partial depletion of *Taf5*, *Taf7*, *Cdk7* or *CycH* (Fig 9B and 9C). The Trx-induced ectopic expression of *Abd-B* was also sensitive to knockdown of *CycH* (Figs 9D and S18A), whereas the orthotopic expression of *Antp* was not (Figs 9E, 9F and S18B). Interestingly, the ectopic expression domain of *Abd-B* extended to the ventral compartment by over-expressing *CycH* (Figs 9G and S18A). As a control, over-expression of *CycH* alone did not induce ectopic expression of *Abd-B* (Figs 9H and S18A) or affect the orthotopic express of *Antp* (Figs 9I and S18C). Collectively, we propose that ectopic gene expression involves more sensitive coordination between H3.3 deposition, TFII-D, and TFII-H activities than is required for orthotopic gene expression.

## Discussion

In this study, we provide evidence that loss of *dBRWD3* suppresses ectopic gene expression and the tissue proliferation caused by the loss of PcG function, but not orthotopic gene expression. Loss of *dBRWD3* also suppresses the ectopic gene expression induced by Trx. This suppression is related to enhanced H3.3 deposition at transcription start sites and reduced H3K4me3, activated PolII, and total PolII levels around the 5' regions of the ectopically expressed genes.

### Excessive histone H3.3 deposition negatively affects ectopic gene transcription

A genome-wide H3.3 ChIP study revealed that H3.3 is enriched in enhancers, promoters, and gene bodies of actively transcribed genes [48]. This correlation suggests that H3.3 deposition may promote gene transcription, a concept that has been supported by the fact that H3.3s are more likely to possess marks associated with active gene expression, including trimethylation at lysine 4 (H3.3K4me3) and acetylation at several lysine residues [49,50]. However, an elegant study demonstrated that gene transcription remains normal when H3.3 is replaced by H3.3K4A, casting doubt regarding the importance of H3.3K4me3 [51]. Moreover, the extent to which H3.3 deposition truly promotes gene transcription is difficult to determine from genetic studies because knockout of H3.3 concurrently leads to up-regulation of one set of genes and down-regulation of another[52]. In the case of trans-retinoid acid induced expression of *Cyp26A1* in embryonic stem cells, H3.3 is actively deposited to the enhancer before induction. Upon induction, H3.3 is depleted from the enhancer but deposited into the promoter. Knockdown of H3.3 reduces the binding of RAR and Tip60 to the enhancer region, indicating that deposition of H3.3 at enhancer regions facilitates the activation of inducible genes [53]. However, the role of H3.3 at promoter and gene body in transcription remain unclear.

It is even less clear how H3.3 affects ectopic gene expression, a pathological condition frequently associated with various cancers in humans [54,55]. In this study, we provide several lines of evidence that show that the regulation of H3.3 by *dRBWD3* is required for the ectopic gene expression observed in *PcG* mutants or upon over-expression of *trx*. Firstly, only *dBRWD3* transgenes that are able to reduce H3.3 levels in *dBRWD3* mutant cells are capable of restoring ectopic expression of *upd3* in *Scm*, *dBRWD3* double-mutant cells. Secondly, the loss of *yem*, which prevents the aberrant incorporation of H3.3, also restores the ectopic expression of *upd3* in *Scm*, *dBRWD3*, *yem* triple-mutant clones. Conversely, the over-deposited H3.3 induced by YEM is sufficient to suppress the ectopic *Antp* expression and Trx-induced ectopic *Abd-B* expression.

In ectopic gene expression, H3.3 deposition at the enhancers is variably regulated by dBRWD3. Nevertheless, through a not entirely clear mechanism, the H3K4me1 induced by Trx at enhancers is insensitive to dBRWD3. By contrast, the *dBRWD3* depletion-enhanced H3.3 deposition at transcription start sites interferes with H3K4me3 and PolII enrichment at the same regions as well as the 5' ends of the gene bodies. Taken together, these observations suggest that H3.3 deposition at these regions disrupts transcription by interfering with trimethylation of H3K4 as well as PolII engagement or activation. This notion is supported by a recent finding that ectopic gene expression persists longer in *Hira* mutants, in which H3.3 deposition is reduced at the promoter and 5' regions [56]. Different from the known role of H3K4me3 on the promoters, our data shows that H3K4me3 is enriched on the gene bodies of ectopically or orthotopically expressed genes rather than the promoters. This is most likely due to the bias associated with the selected PCR amplicons.

dBRWD3 regulates the deposition of H3.3 more prominently at the promoters and transcription start sites in both ectopic and orthotopic gene expression. However, dBRWD3 regulates the PolII occupancy and H3K4me3 levels only in ectopic gene expression. Due to a not-yet-defined “robustness” of transcription, orthotopic gene expression is rendered insensitive to the increase of H3.3. Our data suggest that the same robustness of orthotopic gene expression closely cooperates with TFII-D and TFII-H since orthotopic gene expression remains intact under the suboptimal TFII-D or TFII-H activities. Further studies are needed to understand molecular nature of the robustness.

In addition to their negative effects on ectopic gene expression, H3.3 deposition and dBRWD3 may also interact with PcG in different contexts. For example, it has been reported that H3.3 deposition directs PRC2 to bivalent promoters in ES cells [57]. In addition, loss of dBRWD3 up-regulates *Pc*, *pho*, and *tna* but down-regulates *phol*, *Jarid2* and the trithorax group genes *ash1* and *Iswi* [11]. The regulation of *ash1*, which encodes H3K4 monomethylase, is particularly interesting because an independent transcriptome analysis of adult heads in *dBRWD3* hypomorphic mutants also confirmed a lower level of *ash1* mRNA. However, functional studies revealed that *ash1* depletion by RNAi rescues rather than exacerbates the rough eye phenotype caused by *dBRWD3* depletion. In addition, the H3K4me1 levels in *dBRWD3* mutant cells are similar to those in *wild-type* cells. Further studies are needed to determine the significance of dBRWD3 regulation of *ash1* mRNA. Finally, knockdown of *dBRWD3* causes further reduction of H3K27me3 or H2AK118ub in the *E(z)* or *Pc* depleted brains, suggesting that H3.3 deposition may accelerate the removal of these repressive marks. Further investigations are warranted to understand whether the accelerated removal of repressive marks is mainly contributed by the nucleosome turnover associated with H3.3 deposition or involves activation of demethylases and deubiquitylase.

### Biological implications of differences between ectopic and orthotopic gene expression

The H3K4me3 levels at the promoter and 5' ends of genes correlate well with active transcription. In fact, it is both a cause and a consequence of active transcription. As a cause, H3K4me3 recruits the TFII-D subunit TAF3, a general transcription factor involved in PolII engagement and transcription initiation [58]. Based on such a scenario, we propose that dBRWD3 increases H3K4me3 levels to the extent required for promoters to recruit TFII-D in ectopic gene expression. Consistently, partial knockdown of *Taf5* or *Taf7* affects ectopic but not orthotopic gene expression. In other words, ectopic and orthotopic gene expression may require different levels of TFII-D activities.

During active transcription, H3K4me3 is established by hSet1A/B, which is recruited to actively transcribed gene regions by CTD Ser5-phosphorylated PolII [59]. The phosphorylation of PolII’s CTD Ser5 is mediated by CDK7/CycH and is preferentially required for ectopic gene expression. Consistent with this idea, we demonstrated that partial knockdown of *Cdk7* or *CycH* indeed affected ectopic gene expression without a discernible effect on orthotopic gene expression, perhaps because it was more dependent on the phosphorylation of PolII CTD Ser5. A complement study in RING1A, RING1B double knockout embryonic stem cells revealed that de-repressed loci displaying higher levels of PolII phospho-CTD Ser5 are ectopically expressed at higher levels [60], supporting our findings that phosphorylation of PolII CTD Ser5 plays an unique role in ectopic gene expression that is not shared with orthotopic gene expression. Based in part on these findings, we propose that dBRWD3 could play a preferential role in ectopic gene expression by facilitating the phosphorylation of PolII CTD Ser5.

*PcG* mutations and reduced expression of PcG proteins contribute to tumorigenesis in several human malignancies [25–31,34,35,61–64]. Hence, understanding the regulation of ectopic gene expression will have important medical implications. It has been shown that the ectopic expression of *upd1*, *upd2*, and *upd3* also underlies tissue overgrowth in *Drosophila PcG* mutants, suggesting an evolutionarily conserved role for PcG in tumor suppression from insects to humans. Based on our results, we speculate that inhibition of the BRWD3, TFII-D, and TFII-H complex, for example by the CDK7 inhibitor THZ1 [65–67], might preferentially suppress a broad spectrum of tumors driven by *PcG* mutations.

In summary, we found that ectopic gene expression differs from orthotopic gene expression in their sensitivities to dBRWD3. Inactivation of dBRWD3 selectively suppresses ectopic gene expression and tissue overgrowth induced by loss of PcG function.

## Materials and Methods

### Constructs

*p-ENTR-dBRWD3-N1287A-3XFlag*, *p-ENTR-dBRWD3-N1451A-3XFlag*, *p-ENTR-dBRWD3-N1287A*, *N1451A-3XFlag* (*p-ENTR-dBRWD3-2BC-3XFlag*) were generated with the Thermo Scientific Phusion Site-Directed Mutagenesis kit using the previously described *p-ENTR-dBRWD3-3XFlag* as a template. *p-ENTR-HA-yem* was generated by PCR from the cDNA clone RE33235, Drosophila Genetic Resource Center. *p-ENTR-dBRWD3-N1287A-3XFlag*, *p-ENTR-dBRWD3-N1451A-3XFlag*, and *pENTR- dBRWD3-2BC-3XFlag* were recombined into the *pUWR* vector (DGRC Gateway collection) to generate *pUWR-dBRWD3-N1287A-3XFlag-RFP*, *pUWR-dBRWD3-N1451A-3XFlag-RFP*, *pUWR-dBRWD3-2BC-3XFlag-RFP*. *p-ENTR-HA-yem* was recombined into the *pTWF* vector (DGRC Gateway collection) to generate *pTWF-HA-yem*.

### Fly strains and genetics

Flies were raised in standard conditions at 25°C except as otherwise mentioned. The *dBRWD3*^*s5349*^, and *yem*^*GS21861*^ were described earlier [11,68]. *Sce*^*KO*^ and *Scm*^*D1*^, *trx*^*E2*^ were kindly provided by Dr. Muller [39,47]. *hs-H3*.*3-GFP* was a gift from Dr. Kami Ahmad [69]. *GMR-GAL4* (stock number 9146), *Elav-GAL4* (stock number 458), *Scm*^*D1*^ (stock number 24158), *Sce*^*1*^ (stock number 24618), *wts*^*x1*^ (stock number 44251), *ex-LacZ* (stock number 11067), *UAS-mCD8-GFP* (stock number 5146), *UAS-Taf5-shRNA* (stock number 35367), and *UAS-Taf7-shRNA* (stock number 55216) were obtained from the Bloomington stock center. *UAS-trx* (stock number 12194) and *OK107-GAL4*, were obtained from the Drosophila Genetic Resource Center, Kyoto. The *Scm*^*D1*^, *dBRWD3*^*s5349*^ double mutant, *Scm*^*D1*^, *dBRWD3*^*PX2*^ double mutant, *dBRWD3*^*s5349*^, *Sce*^*1*^ double mutant, *Scm*^*D1*^, *dBRWD3*^*s5349*^, *yem*^*GS21861*^ triple mutant, and *wts*^*x1*^, *dBRWD3*^*s5349*^ double mutant were generated by recombination. *UAS-Psc-dsRNA* (NIG3886R-4) *UAS-ph-p-dsRNA* (NIG18412R-1), *UAS-E(z)-dsRNA* (NIG6502R-3), *UAS-Pc-dsRNA* (NIG32443R-1), *UAS-CycH-dsRNA* (NIG7405R-1), and *UAS-Cdk7-dsRNA* (NIG3319R-1) were obtained from the fly stocks of the National Institute of Genetics, Kyoto, Japan (NIG-FLY). *UAS-dBRWD3-dsRNA* (VDRC40209) was obtained from the Vienna Drosophila RNAi Center (VDRC). The transgenic flies *ubi-dBRWD3-N1287A-3XFlag-RFP*, *ubi-dBRWD3-N1451A-3XFlag-RFP*, *ubi-dBRWD3-N1287A*, *N1451A-3XFlag-RFP* (*ubi-dBRWD3-2BC-3XFlag-RFP*), and *pTWF-HA-yem* were generated by microinjection for germ-line transformation. The transgenic flies *ubi-H3*.*3-dendra2*, *ubi-dBRWD3-3XFlag-RFP*, *ubi-delta-N-dBRWD3-3XFlag-RFP*, and *10XSTAT-nlsGFP* were described previously [11,41].

### Clonal analysis

Genotypes for mosaic mutant clones in eyes:

1. *ey-flp/+*; *FRT*^*82B*^ */FRT*^*82B*^ *ubi-nlsGFP* (Figs 1B, 3A, 3B, 3C, 3G, 4C and 4K)
2. *ey-flp/+*; *FRT*^*82B*^ *Scm*^*D1*^*/FRT*^*82B*^ *ubi-nlsGFP* (Figs 1C, 3A, 3B, 3C, 3E, 4A and 4J)
3. *ey-flp/+*; *FRT*^*82B*^ *Scm*^*D1*^, *dBRWD3*^*s5349*^*/FRT*^*82B*^ *ubi-nlsGFP* (Figs 1D, 3A, 3B, 3C, 3F, 4F and 4L)
4. *ey-flp/+*; *FRT*^*82B*^ *Sce*^*1*^*/FRT*^*82B*^ *ubi-nlsGFP* (Figs 1E, 3H, 4B and 4O)
5. *ey-flp/+*; *FRT*^*82B*^ *dBRWD3*^*s5349*^, *Sce*^*1*^*/FRT*^*82B*^ *ubi-nlsGFP* (Figs 1F, 3I, 4G and 4N)
6. *hs-flp/+*; *FRT*^*82B*^ */FRT*^*82B*^ *ubi-nlsGFP* (Fig 1H)
7. *hs-flp/+*; *FRT*^*82B*^ *dBRWD3*^*s5349*^*/FRT*^*82B*^ *ubi-nlsGFP* (Fig 1I)
8. *ey-flp/+*; *FRT*^*82B*^ *dBRWD3*^*s5349*^*/FRT*^*82B*^ *ubi-nlsGFP* (Figs 3J and 4H)
9. *ey-flp/+*; *10XSTAT-nlsGFP/+*; *FRT*^*82B*^ *Scm*^*D1*^*/FRT*^*82B*^ *ubi-mof-RFP* (Fig 3N)
10. *ey-flp/+*; *10XSTAT-nlsGFP/+*; *FRT*^*82B*^ *Scm*^*D1*^, *dBRWD3*^*s5349*^*/FRT*^*82B*^ *ubi-mof-RFP* (Fig 3O)
11. *ey-flp/+*; *10XSTAT-nlsGFP/+*; *FRT*^*82B*^ *dBRWD3*^*s5349*^*/FRT*^*82B*^ *ubi-mof-RFP* (Fig 3P)
12. *ey-flp/+*; *FRT*^*82B*^ *wts*^*x1*^*/FRT*^*82B*^ *ubi-nlsGFP* (Fig 4Q)
13. *ey-flp/+*; *FRT*^*82B*^ *dBRWD3*^*s5349*^, *wts*^*x1*^*/FRT*^*82B*^ *ubi-nlsGFP* (Fig 4R)
14. *ey-flp/+*; *ubi-dBRWD3-RFP/+*; *FRT*^*82B*^ *Scm*^*D1*^, *dBRWD3*^*s5349*^*/FRT*^*82B*^ *ubi-nlsGFP* (Fig 5A)
15. *ey-flp/+*; *ubi-dBRWD3-2BC-RFP/+*; *FRT*^*82B*^ *Scm*^*D1*^, *dBRWD3*^*s5349*^*/FRT*^*82B*^ *ubi-nlsGFP* (Fig 5B)
16. *ey-flp/+*; *ubi-△N-dBRWD3-RFP/+*; *FRT*^*82B*^ *Scm*^*D1*^, *dBRWD3*^*s5349*^*/FRT*^*82B*^ *ubi-nlsGFP* (Fig 5C)
17. *ey-flp/+*; *FRT*^*82B*^ *Scm*^*D1*^, *dBRWD3*^*s5349*^, *yem*^*GS21861*^*/FRT*^*82B*^ *ubi-nlsGFP* (Fig 5D and 5E)
18. *ey-flp/GMR-GAL4*; *UAS-HA-yem-Flag*, *FRT*^*82B*^ *Scm*^*D1*^*/FRT*^*82B*^ *ubi-nlsGFP* (Fig 5G)

### RNA extraction, reverse transcription and RT-PCR

Total RNA was isolated from instar larval mosaic eye brain complexes using TRIzol reagent (Invitrogen). Following the manufacturer’s protocol, cDNA was synthesized using oligo(dT) and SuperScript reverse transcriptase (Invitrogen). OmicsGreen qPCR 5X Master Mix (Omics Bio) was used for real-time quantitative PCR on a CFX96 connect Real-Time PCR System (Bio-Rad). RPL32 was used as an endogenous loading control.

### Immunostaining and antibodies

3^rd^ instar larval eye imaginal discs were dissected in PBS and fixed for 17 minutes in 4% formaldehyde, followed by three 10-min washes in PBS supplemented with 0.3% Triton-X-100 (PBT) and 30-min blocking in PBT containing 5% normal donkey serum (NDS). After blocking, discs were incubated with primary antibody either overnight at 4°C or 2 hours at room temperature in PBT containing 5% NDS. After incubation with primary antibody, discs were washed three times in PBT before incubating with secondary antibody in PBT containing 5% NDS for one hour at room temperature. After three subsequent washes, discs were mounted with glycerol. Primary antibodies used in this study include mouse anti-H2AK118ub (1:100, Millipore, E6C5), rabbit anti-H3K27me3 (1:100, Millipore), rabbit anti-H3K4me1 (1:100, Active Motif), rabbit anti-H3S10ph (1:500, Millipore), mouse anti-Ubx (1:20, DSHB, Ubx), mouse anti-Antp (1:20, DSHB, 8C11), rabbit anti-upd3 (1:750), and mouse anti-β-Galactosidase (1:1000, Sigma, GAL-50). Secondary antibodies include goat anti-mouse Cy3 (1:1000, Jackson ImmunoResearch), goat anti-mouse Cy5 (1:1000, Jackson ImmunoResearch), and goat anti-rabbit Cy3 (1:1000, Jackson ImmunoResearch).

### Chromatin immunoprecipitation

Chromatin immunoprecipitation was done with a ChIP-IT High Sensitivity (HS) Kit (Active Motif) following the instructions provided by the manufacturer. Briefly, 300 pairs of brain lobes (leaving out the attached ventral nerve cords) or wing imaginal discs of 3rd instar larvae were collected. The collected tissues were fixed with complete tissue fixation solution (28μl 37% formaldehyde in 970μl PBS) at room temperature for 15 minutes. Fixation was stopped with the stop solution at room temperature for 5 minutes. The fixed tissues were washed with ice-old PBS wash buffer and then immersed in the tissues with the chromatin prep buffer. The fixed tissues were sonicated using the UP50H Ultrasonic Processor (Hielscher-Ultrasound Technology), with 30% amplitude and 20 pulse cycles of 30 seconds on followed by 30 seconds off. 6 μg sheared chromatin was incubated with 1μg antibodies overnight at 4°C. 30 μl Protein G agarose beads were added to each IP reaction. The mixture was rotated at 4°C for 3 hours. The ChIP reactions were loaded into columns and washed. The ChIP DNA was obtained by eluting the columns with elution buffer AM4. The elute was treated with Protease K at 55°C for 30 minutes, 80°C for two hours, followed by column clean-up. OmicsGreen qPCR 5X Master Mix (Omics Bio) was used for real-time quantitative PCR on a CFX96 connect Real-Time PCR System (Bio-Rad) to measure the amount of ChIP DNA and input DNA containing indicated sequences from enhancers, promoters, and 5' transcription regions of *Antp*, *Ubx*, and *Abd-B*. Primary antibodies used include rabbit anti-H2AK118ub (Cell signaling, D27C4), mouse anti-H3K27me3 (Abcam, mAbcam 6002), rabbit anti-H3K4me1 (Abcam, ab8895), rabbit anti-H3K4me3 (Abcam, ab8580), mouse anti-PolII (Abcam, 4H8), and rabbit anti-PolII phospho-CTD Ser5 (Abcam, ab5131), rabbit anti-HA (Cell Signaling, C29F4) and mouse anti-Flag (Sigma, M2)

### Image quantification

All confocal images were obtained by LSM 700 laser scanning confocal microscope (Carl Zeiss). For quantitative analysis of protein levels, the antibody staining conditions, laser power, and pinhole sizes were kept identical among groups. Pixel number, pixel intensity, and area were provided by the built-in software in LSM 700. The areas of clones (marked by the absence of GFP) and non-clones (marked by GFP) were calculated by the total GFP positive and GFP negative areas respectively. Antp-positive regions in ventral nerve cords were manually marked. The Antp expression areas and lengths were calculated by the built-in software according to the marked regions. H3S10ph-positive mitotic cells and Antp-positive brain cells were manually counted.

## Supporting Information

S1 Text Additional information on primers for RT-qPCR and genotypes of clonal analyses shown in Supporting Information figures. (DOCX)

S1 Fig (A) Antp levels (arrows) in *Scm*^*D1*^, *dBRWD3*^*PX2*^ double-mutant clones generated in the 3^rd^ instar eye imaginal discs by *ey-flp* and marked by the absence of GFP. Scale bars indicate 50μm. (B) A schematic illustration of how protein levels are calculated from a confocal image of a mosaic imaginal disc. (C and D) Quantifications of *Antp* expression. The ectopic expression of *Antp* in *Scm*^*D1*^, *Scm*^*D1*^, *dBRWD3*^*PX2*^ and *Scm*^*D1*^, *dBRWD3*^*s5349*^ mutant clones (C), *Sce*^*1*^ and *dBRWD3*^*s5349*^, *Sce*^*1*^ mutant clones (D). (E)The orthotopic expression of *Antp* in wild type and *dBRWD3*^*s5349*^ mutant clones. a.u. indicates arbitrary unit. Data are shown as means ± S.D. *, **, *** indicate P< 0.01, 0.001, 0.0001, respectively, by Student's t-test. ns. indicates not significant.
(TIF)

S2 Fig (A) The knockdown efficiency of *Pc-dsRNA* driven by *Elav-GAL4*. (B-D) The mean intensity (B), expression area (C), and length of expression domain (D) of *Antp* in control, *Pc* depleted, *dBRWD3* depleted, and *Pc*, *dBRWD3* doubly depleted ventral nervous cords. (E) The knockdown efficiency of *E(z)-dsRNA* driven by *Elav-GAL4*. (F-H) The mean intensity (F), expression area (G), and length of expression domain (H) in the control, *E(z)* depleted, *dBRWD3* depleted, and *E(z)*, *dBRWD3* doubly depleted ventral nervous cords. a.u. indicates arbitrary unit. Data are shown as means ± S.D. ns. indicates P>0.05 by Student's t-test. *, *** indicate P< 0.01, 0.0001, respectively, by Student's t-test.
(TIF)

S3 Fig (A and B) Quantification of Upd3 ectopic expression in *Scm*^*D1*^ and *Scm*^*D1*^, *dBRWD3*^*s5349*^ mutant clones (A) or *Sce*^*1*^ and *dBRWD3*^*s5349*^, *Sce*^*1*^ mutant clones (B). Data are shown as means ± S.D. *, **, *** indicate P< 0.01, 0.001, 0.0001, respectively, by Student's t-test. ns. indicates not significant. a.u. indicates arbitrary unit.
(TIF)

S4 Fig Upd3 levels (arrows) in *Scm*^*KO*^ mutant clones generated in 3^rd^ instar eye imaginal discs by *ey-flp* and marked by the absence of GFP. Scale bar indicates 50μm.
(TIF)

S5 Fig *dBRWD3* does not regulate the activation of hippo pathway downstream genes. (A and B) *expanded-LacZ* (*ex-LacZ*) expression (arrows) in *wts*^*x1*^ mutant clones (A), and *dBRWD3*^*s5349*^, *wts*^*x1*^ double-mutant clones (B). Scale bars indicate 50μm.
(TIF)

S6 Fig *dBRWD3 BRD1* or *BRD2* mutant supports the ectopic expression of *upd3*. (A) A diagram illustrating the molecular structure of dBRWD3. (B and C) upd3 levels (arrows) in *Scm*^*D1*^, *dBRWD3*^*s5349*^ double-mutant clones complemented with ubiquitously expressed *dBRWD3-N1287A-RFP* (B) or *dBRWD3-N1451A-RFP* (C). Scale bars indicate 50μm.
(TIF)

S7 Fig The expression of indicated *dBRWD3* transgenes measured by RT-qPCR in the presence of endogenous *dBRWD3*. Data represent the mRNA levels of indicated *dBRWD3* transgenes plus endogenous *dBRWD3* and are shown in means ± S.D., n = 4. *, **, *** indicate P< 0.01, 0.001, 0.0001 respectively in comparison to that in *Canton-S* by student's t-test. a.u. indicates arbitrary unit.
(TIF)

S8 Fig H3.3 levels regulated by various *dBRWD3* transgenes. (A-D) H3.3-dendra2 driven by a *ubi*-promoter was expressed in *dBRWD3*^*s5349*^ mosaic mutant eye discs. *dBRWD3*^*s5349*^ mutant clones were marked by the absence of RFP (arrows), with concomitant expression of *ΔN-*dBRWD3-RFP (A), dBRWD3-2BC-RFP (B), *dBRWD3-N1287A-RFP* (C), and *dBRWD3-N1451A-RFP* (D). Scale bars indicate 50μm. (E) The levels of endogenous H3.3 in *dBRWD3*-depleted brains and ventral nerve cords.
(TIF)

S9 Fig The levels of H3.3-dendra2 accumulation inversely correlate with ectopic gene expression. (A) H3.3-dendra2 and Antp levels in *wild type* eye discs. (B-D) H3.3-dendra2 and Antp levels in *Scm*^*D1*^, *dBRWD3*^*s5349*^, double-mutant clones (B), *Scm*^*D1*^, *dBRWD3*^*s5349*^, *yem*^*GS21861*^ triple-mutant clones (C), and *Scm*^*D1*^ single-mutant clones (D) that were generated in the eye disc and marked by the absence of RFP (arrows). Scale bars indicate 50μm.
(TIF)

S10 Fig Ectopic gene expression is regulated by YEM activity. (A and B) The mean intensity of Antp (A) and Upd3 (B) staining in *wild type* and *Scm*^*D1*^ *dBRWD3*^*s5349*^, *yem*^*GS21861*^ triple-mutant clones. *** indicates p<0.0001 by Student's t-test. (C) The mean intensity of Antp staining in *Scm*^*D1*^ and *Scm*^*D1*^ *UAS*-*yem*. *** indicates p<0.0001 by Student's t-test.
(TIF)

S11 Fig Over-expression of *yem* enhances H3.3 deposition. (A and B) Heat-shock inducible H3.3-GFP was expressed in *wild-type* (A) and *yem-*expressing (B) salivary glands. (C) The quantification of H3.3-GFP levels in (A) and (B).
(TIF)

S12 Fig *dBRWD3* does not regulate the removal of pre-existing H3K27me3 and H2AK118ub. (A) A schematic illustration of the expression domain of *ms1096-GAL4*. Expression in the dorsal compartment (dark grey) is higher than in the ventral compartment (light grey). (B-F) Immunofluorescence studies of H3K27me3 levels in the *E(z)*, *dBRWD3* doubly depleted (B), *E(z)* depleted (C), *Pc*, *dBRWD3* doubly depleted (D), *Pc* depleted (E), and control (F) wing discs. (G and H) H2AK118ub levels in *Sce*^*KO*^ (G) and *dBRWD3*^*s5349*^, *Sce*^*KO*^ (H) mutant clones. Scale bars indicate 50μm.
(TIF)

S13 Fig *Scm* mutation induced ectopic gene expression depends on *trx*. The ectopic expression of *Antp* in *Scm*^*D1*^, *trx*^*E2*^ double-mutant clones marked by the absence of GFP (arrows). Scale bars indicate 50μm.
(TIF)

S14 Fig *dBRWD3* suppresses ectopic gene expression caused by overexpression of *trx*. (A) A schematic illustration of the disc proper and the peripodial epithelium of the eye disc. (B and C) Antp levels in the *trx*-expressing (B) and *trx*-expressing, *dBRWD3*-depleted (C) peripodial epithelia. Scale bars indicate 50μm. (D-E) *trx* was overexpressed under the control of *GMR-GAL4*. The TRX-induced Antp expression (arrows) in *wild-type* (D), *dBRWD3* depletion (E) backgrounds. (F-G) *trx* was overexpressed under the control of *ms-1096-GAL4*. The TRX-induced Ubx expression (arrows) in *wild-type* (F), *dBRWD3* depletion (G) backgrounds. Scale bars indicate 20μm.
(TIF)

S15 Fig *dBRWD3* does not regulate the methyltransferase activity of Trx. (A) A schematic illustration of the *apterous-GAL4* expression region in the 3^rd^ wing imaginal discs. D indicates the dorsal compartment. (B-D) Immunofluorescence studies of H3K4me1 levels in the dorsal compartment of the *apterous-GAL4* control (B), *trx*-expressing (C), and *trx*-expressing, *dBRWD3* depleted (D) wing discs marked by GFP. Scale bars indicate 50μm. (E) A quantitative analysis of H3K4me1 levels in control, *trx* over-expression, and *trx* over-expression, *dBRWD3*-depleted wings. *** indicates p<0.0001 by Student's t-test. ns. indicates not significant.
(TIF)

S16 Fig Knockdown of *dBRWD3* suppressed only the Trx-induced increase of PolII occupancy but not the PolII occupancy of orthotopically expressing *Antp*. A ChIP-qPCR analysis of PolII levels at the promoters and transcription start sites of *Antp* in the *UAS-mCD8-GFP* control, in wings over-expressing *trx*, or in wings with concurrent *trx* over-expression and *dBRWD3* depletion. ns. indicates not significant. ChIP-qPCR Data are shown as means ± S.D from 4 technical replicates. *, ** indicate P<0.05, 0.01 respectively by Student's t-test.
(TIF)

S17 Fig Partial knockdown of TFII-D and TFII-H subunits. (A-D) A quantitative analysis of the knockdown efficiencies of *Taf5* (A), *Taf7* (B), *Cdk7* (C), and *CycH* (D) RNAi. *** indicates p<0.0001 by Student's t-test.
(TIF)

S18 Fig *CycH* regulates the ectopic expression of *Abd-B* but not orthotopic expression of *Antp*. (A) A quantitative analysis of Abd-B expression in the control, Trx-over-expressing, Trx-over-expressing plus *CycH* knockdown, Trx and CycH-over-expressing, and CycH-over-expressing wings. (B and C) quantitative analyses of orthotopic expression of *Antp* in the *CycH* knockdown (B) and the CycH-over-expressing (C) wings. *** indicates p<0.0001 by Student's t-test. ns. indicates not significant.
(TIF)
