# Supplementary material for: dBRWD3 Regulates Tissue Overgrowth and Ectopic Gene Expression Caused by Polycomb Group Mutations
Source: PLoS Genet. 2016 Sep 2;12(9):e1006262. doi: 10.1371/journal.pgen.1006262 (PMC5010193; doi:10.1371/journal.pgen.1006262)
Supplement: S1 Text — (DOCX) [file pgen.1006262.s001.docx]

**S1 Text**

Additional information on primers for RT-qPCR and genotypes of clonal analyses shown in Supporting Information figures.

**Primers for RT-qPCR**

| Gene symbol | Primer sequence (5’-3’) (F: Forward, R: Reverse) |
| --- | --- |
| *upd1* | F: TGG CCA CGT AAG TTT GCA TGT TG |
|  | R: TAG CCA GGA CTC GGC CAT AGA TAT C |
| *upd2* | F: ATG TGG CGG TAC CAA GTC TTT AGC |
|  | R: TAC AAG TTC CTG CCG AAC ATG AC |
| *upd3* | F: ACA AGT GGC GAT TCT ATA AGG GAA AC |
|  | R: ATG TTG CGC ATG TAC GTG AAG G |
| *RPL32* | F: CCA AGA TCG TGA AGA AGC G |
|  | R: GTT GGG CAT CAG ATA CTG TC |
| *dBRWD3* | F: ACT CCG ACT CGG AAG ACA AT |
|  | R: GCG ATG GAG TTA TTG GAT GG |
| *Ubx promoter* | F: GGC GAG CGC ATT TTC CTT |
|  | R: GCG CTC TCT CTT GAG TGT TCG T |
| *Ubx +7~+96* | F: GTT CGA TGG CAA CGG ATT GG |
|  | R: CGC TGA GGG CCA ATA ATA ACA A |
| *Ubx +128~+217* | F: ATG CCG CGC GGA AAA GTC |
|  | R: TGC TGA CCG AAC GGG CG |
| *Ubx* | F: CAA TCAC ACA TTC TAC CCC TGG AT |
| *+1668~+1737* | R: ACA CTC ACT TTT GGT CGG ATC TTC |
| *Ubx* | F: CCA CAC GAA TCA TTA TCT GAC CCG |
| *+75424~+75504* | R: TTG ATC TGC CGC TCC GTC AG |
| *abx* | F: CGG TGC CAT TAA CTC TCG ATT |
|  | R: CGC ACG GCA CAT TAA ATT CA |
| *pbx* | F: GAA AAC ACA CAA GTG CAG |
|  | R: GGA GAT CCT AAA ACA TGC |
| *bxd* | F: GCC ATA ACG GCAG AAC CAA AG |
|  | R: ATG AGG CCA TCT CAG TCG C |
| *Abd-B promoter* | F: CCC CAC TTA CAC ACG CAC ACT |
|  | R: GCG TTA TGC ACA CGT ACA CTG TAT AC |
| *Abd-B -4 ~+90* | F: CGT CAG TGT AGA TTT GTG CTT CGC |
|  | R: GCT TGC CTC GGC TGT TGT TG |
| *Abd-B +93~+180* | F: CCC TGC AAC TTC GTC GAG GA |
|  | R: CGA ACG AAA CAC TCC CAG TCG |
| *Abd-B* | F: GAA AGA AGA AAC GTT TTT GGA GTC G |
| *+226~+303* | R: CGG CGA CAC GAT GTT TTG AT |
| *Abd-B* | F: CGA GTG TTT CTC CCT CCA GTG T |
| *+1837~+1824* | R: GGT TTT CAA CCC CAA GGA AAG |
| *Abd-B* | F: ATG TGT ATC GAC GCC GCT TG |
| *+6012~+6263* | R: TGC TCG GGG CAA AAC ATT AA |
| *iab7* | F: GGG TGA TCA GCG ATC GTA GTG |
|  | R: GTG GCC ATA TCC TGC TAA CAC T |
| *iab8* | F: GCC AAC CAG AAG GTC GTA AA |
|  | R: GCT TCT CTT GGC GTT TCA TC |
| *Fab7* | F: TCT TCG GGA TGG CAA TAA AC |
|  | R: ACG ATG TCG GAT TCC TGA AC |
| *Fab8* | F: TGG TGG AAG GAG AAA ACT GG |
|  | R: TGC AGC GAG ACA ATA AAA CG |
| *Antp promoter* | F: CAG TTT GCA GCT CAG CGA GC |
|  | R: GAG TGA CTG AGA GAG CTG AGC GAG |
| *Antp +33～+120* | F: ACA CAA CAA AGC ACT CGA GGA CCC |
|  | R: CTG GGA TCC CTC TCG CAC AC |
| *Antp +150~+260* | F: CCG TAG TCG CTA TAG CGG CCA |
|  | R: GAC GAC GGA GAG CGA GCG AA |
| *Antp* | F:TCG CCA ATT TTC GTT GGG CT |
| *+66961～+67065* | R: CAGGTCAGAATCGAATTCAATTTGC |
| *Antp* | F: GCT GGT GGA TCA AAT GTC CG |
| *+89861～+89930* | R: CGG ATG TCC CAT GTG ATG GG |
| *Antp PRE* | F: CAG CTG TCG AGG TGT GTG GC |
|  | R: AAG CCC AGC CCA CTT AGC CT |
| *E(z)* | F: ACC AAG ATC ACG TGC AAG AAC G |
|  | R: TCA GAC GGA GCC ATG AGC AG |
| *Pc* | F: ATC GGG CAA GAT TGG AGT TAC G |
|  | R: TGG CTG GGA GTT ACC TGC TG |
| *Taf5* | F: CAC CAC CCT GTT GAG GCA CA |
|  | R: CGG TAA CCT TGT GAA AGT CCC AC |
| *Taf7* | F: AGC CGC AGT ACA CGC AGC AA |
|  | R: GTG TGG CAT TCT GAA TGG AAG C |
| *Cdk7* | F: CTG AGC ATG CCG TAT TTC GC |
|  | R: TTG AGC GCA GGC TTC GTG TC |
| *CycH* | F: CTA TAA GGA GCG CCT ACG CCG |
|  | R: GTG TCC GAG CTC ACA TCT GCA |

**Clonal analyses**

1) *ey-flp/+*; *FRT^82B^ Scm^D1^*, *dBRWD3^PX2^/FRT^82B^ ubi-nlsGFP* (Figure S1A)

2) *ey-flp/+*; *FRT^82B^ Sce^KO^ /FRT^82B^ ubi-nlsGFP* (Figure S4)

3) *ey-flp/+*; *ex-LacZ/+*; *FRT^82B^ wts^x1^/FRT^82B^ ubi-nlsGFP* (Figure S5A)

4) *ey-flp/+*; *ex-LacZ/+*; *FRT^82B^ dBRWD3^s5349^*, *wts^x1^/FRT^82B^ ubi-nlsGFP* (Figure S5B)

5) *ey-flp/+*; *ubi-dBRWD3-N1287A-RFP /+*; *FRT^82B^ Scm^D1^*, *dBRWD3^s5349^/FRT^82B^ ubi-nlsGFP* (Figure S6B)

6) *ey-flp/+*; *ubi-dBRWD3-N1451A-RFP /+*; *FRT^82B^ Scm^D1^*, *dBRWD3^s5349^/FRT^82B^ ubi-nlsGFP* (Figure S6C)

7) *ey-flp/+*; *ubi-H3.3-dendra2*/+; *FRT^82B^ Scm^D1^*, *dBRWD3^s5349^, ubi-△N-dBRWD3-RFP /FRT^82B^ ubi-mof-RFP* (Figure S8A)

8) *ey-flp/+*; *ubi-H3.3-dendra2*, *ubi-dBRWD3-2BC-RFP/+*; *FRT^82B^ Scm^D1^*, *dBRWD3^s5349^/FRT^82B^ ubi-mof-RFP* (Figure S8B)

9) *ey-flp/+*; *ubi-H3.3-dendra2*, *ubi-dBRWD3-N1287A-RFP/+*; *FRT^82B^ Scm^D1^*, *dBRWD3^s5349^/FRT^82B^ ubi-mof-RFP* (Figure S8C)

10) *ey-flp/+*; *ubi-H3.3-dendra2*, *ubi-dBRWD3-N1451A-RFP/+*; *FRT^82B^ Scm^D1^*, *dBRWD3^s5349^/FRT^82B^ ubi-mof-RFP* (Figure S8D)

11) *ey-flp/+*; *ubi-H3.3-dendra2*/*+; FRT^82B^ Scm^D1^*, *dBRWD3^s5349^/FRT^82B^ ubi-mof-RFP* (Figure S9B)

12) *ey-flp/+*; *ubi-H3.3-dendra2*/*+; FRT^82B^ Scm^D1^*, *dBRWD3^s5349^*, *yem^GS21861^/FRT^82B^ ubi-mof-RFP* (Figure S9C)

13) *ey-flp/+*; *ubi-H3.3-dendra2*/*+; FRT^82B^ Scm^D1^/FRT^82B^ ubi-mof-RFP* (Figure S9D)

14) *ey-flp/+*; *FRT^82B^ Sce^KO^ /FRT^82B^ ubi-nlsGFP* (Figure S12G)

15) *ey-flp/+*; *FRT^82B^ dBRWD3^s5349^*, *Sce^KO^ /FRT^82B^ ubi-nlsGFP* (Figure S12H)

16) *ey-flp/+*; *FRT^82B^ Scm^D1^,trx^E2^/FRT^82B^ ubi-nlsGFP* (Figure S13)
