# Supplementary figures and images for: dBRWD3 Regulates Tissue Overgrowth and Ectopic Gene Expression Caused by Polycomb Group Mutations
Source: PLoS Genet. 2016 Sep 2;12(9):e1006262. doi: 10.1371/journal.pgen.1006262 (PMC5010193; doi:10.1371/journal.pgen.1006262)

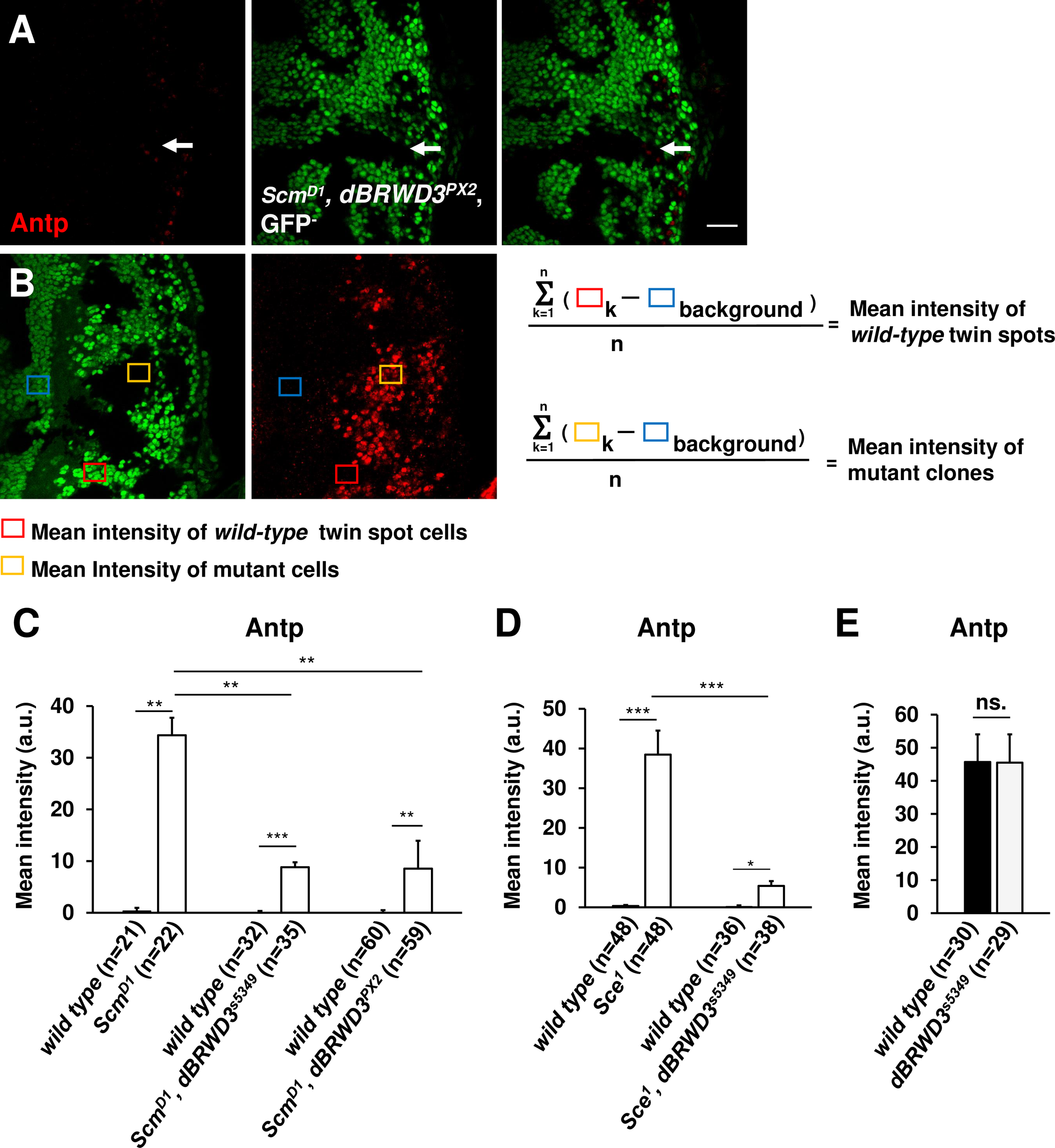

Supplement: S1 Fig — (A) Antp levels (arrows) in ScmD1, dBRWD3PX2 double-mutant clones generated in the 3rd instar eye imaginal discs by ey-flp and marked by the absence of GFP. Scale bars indicate 50μm. (B) A schematic illustration of how protein levels are calculated from a confocal image of a mosaic imaginal disc. (C and D) Quantifications of Antp expression. The ectopic expression of Antp in ScmD1, ScmD1, dBRWD3PX2 and ScmD1, dBRWD3s5349 mutant clones (C), Sce1 and dBRWD3s5349, Sce1 mutant clones (D). (E)The orthotopic expression of Antp in wild type and dBRWD3s5349 mutant clones. a.u. indicates arbitrary unit. Data are shown as means ± S.D. *, **, *** indicate P< 0.01, 0.001, 0.0001, respectively, by Student's t-test. ns. indicates not significant. (TIF) [file pgen.1006262.s002.tif]

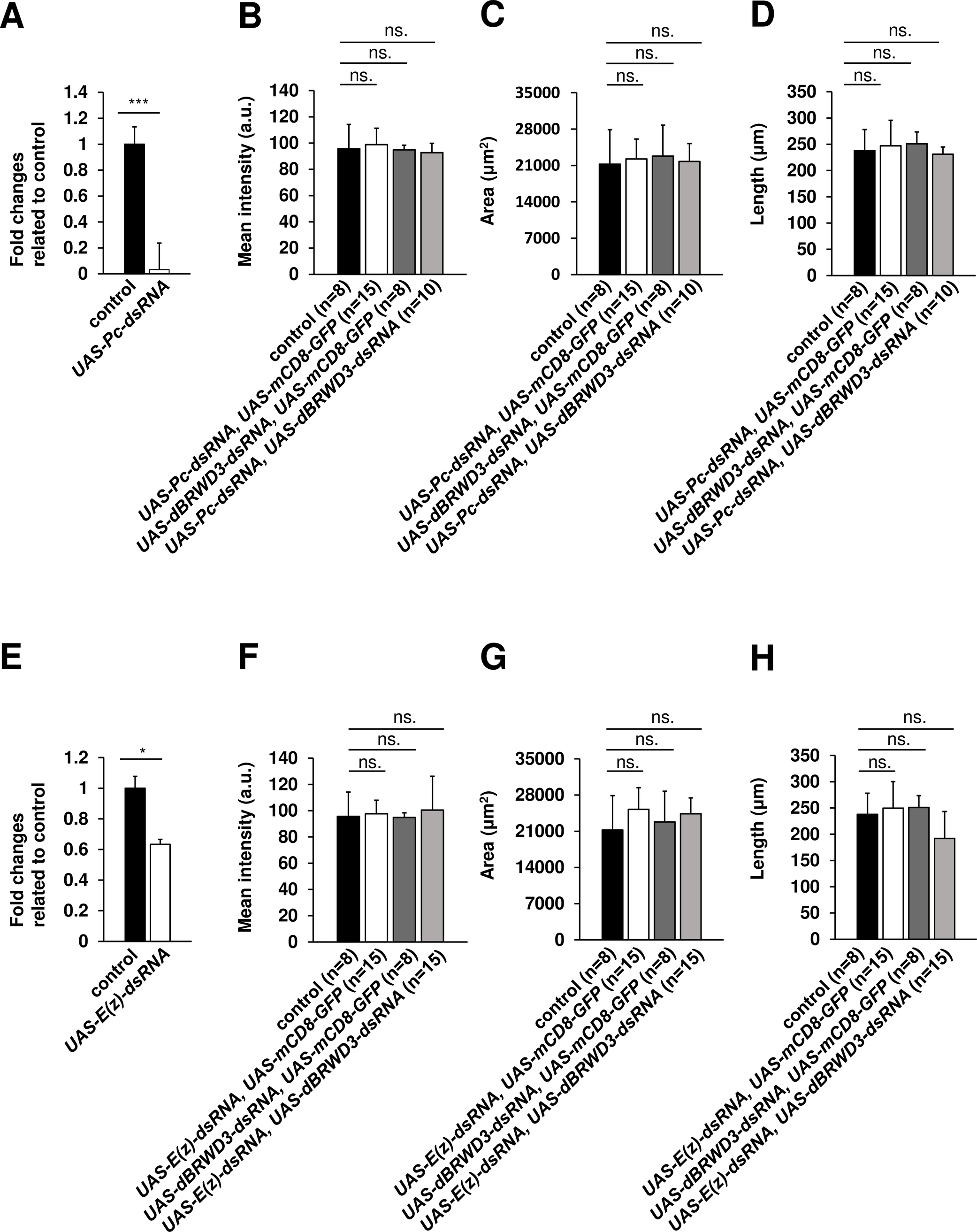

Supplement: S2 Fig — (A) The knockdown efficiency of Pc-dsRNA driven by Elav-GAL4. (B-D) The mean intensity (B), expression area (C), and length of expression domain (D) of Antp in control, Pc depleted, dBRWD3 depleted, and Pc, dBRWD3 doubly depleted ventral nervous cords. (E) The knockdown efficiency of E(z)-dsRNA driven by Elav-GAL4. (F-H) The mean intensity (F), expression area (G), and length of expression domain (H) in the control, E(z) depleted, dBRWD3 depleted, and E(z), dBRWD3 doubly depleted ventral nervous cords. a.u. indicates arbitrary unit. Data are shown as means ± S.D. ns. indicates P>0.05 by Student's t-test. *, *** indicate P< 0.01, 0.0001, respectively, by Student's t-test. (TIF) [file pgen.1006262.s003.tif]

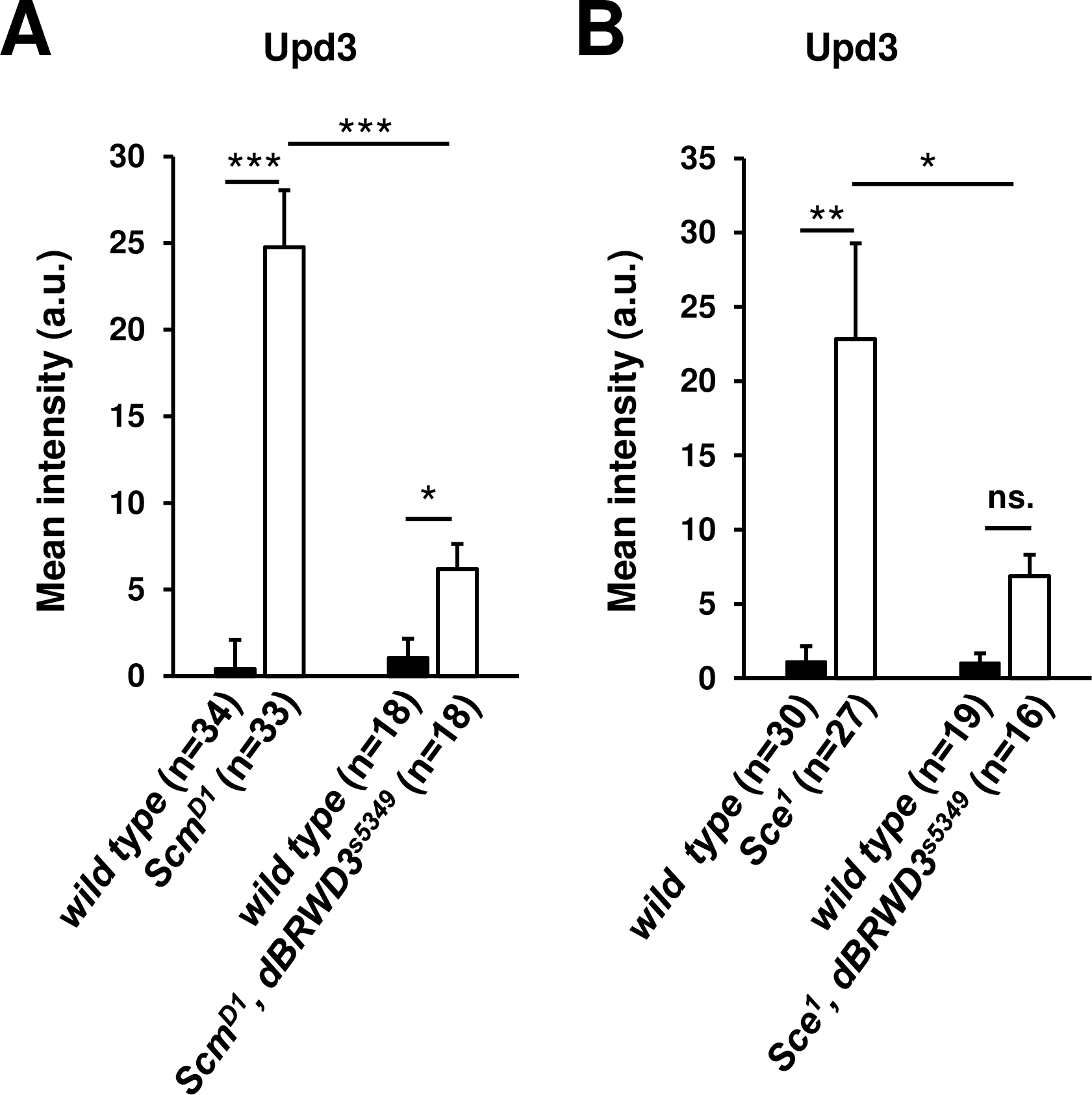

Supplement: S3 Fig — (A and B) Quantification of Upd3 ectopic expression in ScmD1 and ScmD1, dBRWD3s5349 mutant clones (A) or Sce1 and dBRWD3s5349, Sce1 mutant clones (B). Data are shown as means ± S.D. *, **, *** indicate P< 0.01, 0.001, 0.0001, respectively, by Student's t-test. ns. indicates not significant. a.u. indicates arbitrary unit. (TIF) [file pgen.1006262.s004.tif]

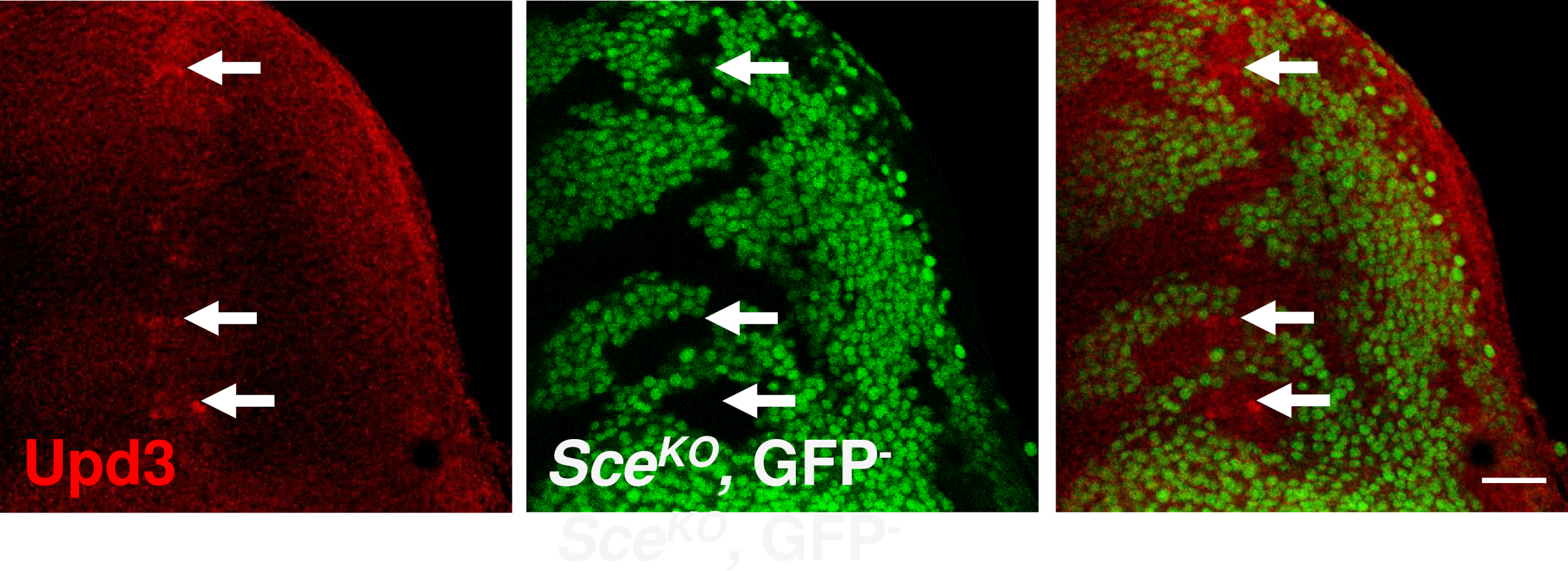

Supplement: S4 Fig — Scale bar indicates 50μm. (TIF) [file pgen.1006262.s005.tif]

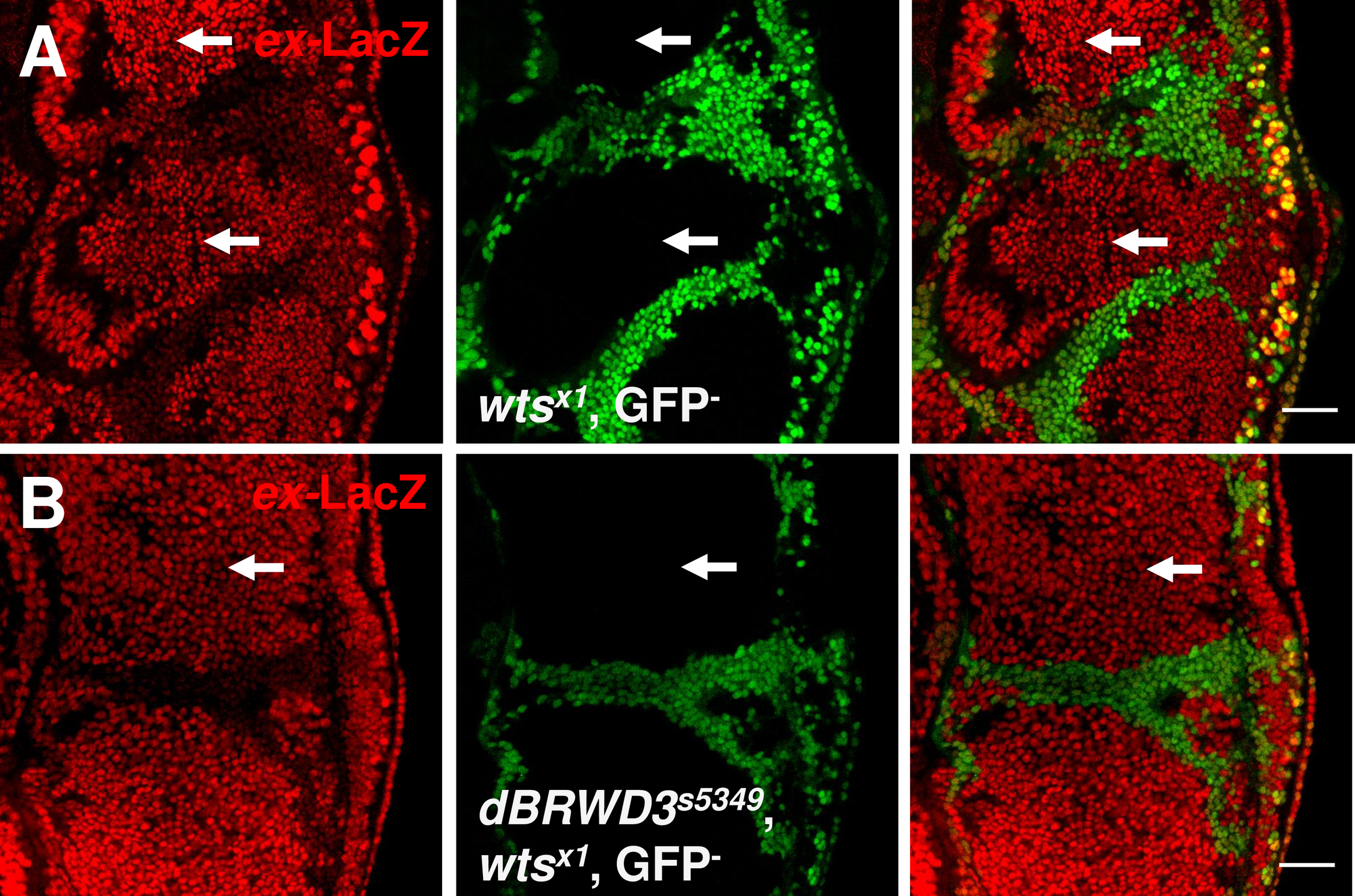

Supplement: S5 Fig — (A and B) expanded-LacZ (ex-LacZ) expression (arrows) in wtsx1 mutant clones (A), and dBRWD3s5349, wtsx1 double-mutant clones (B). Scale bars indicate 50μm. (TIF) [file pgen.1006262.s006.tif]

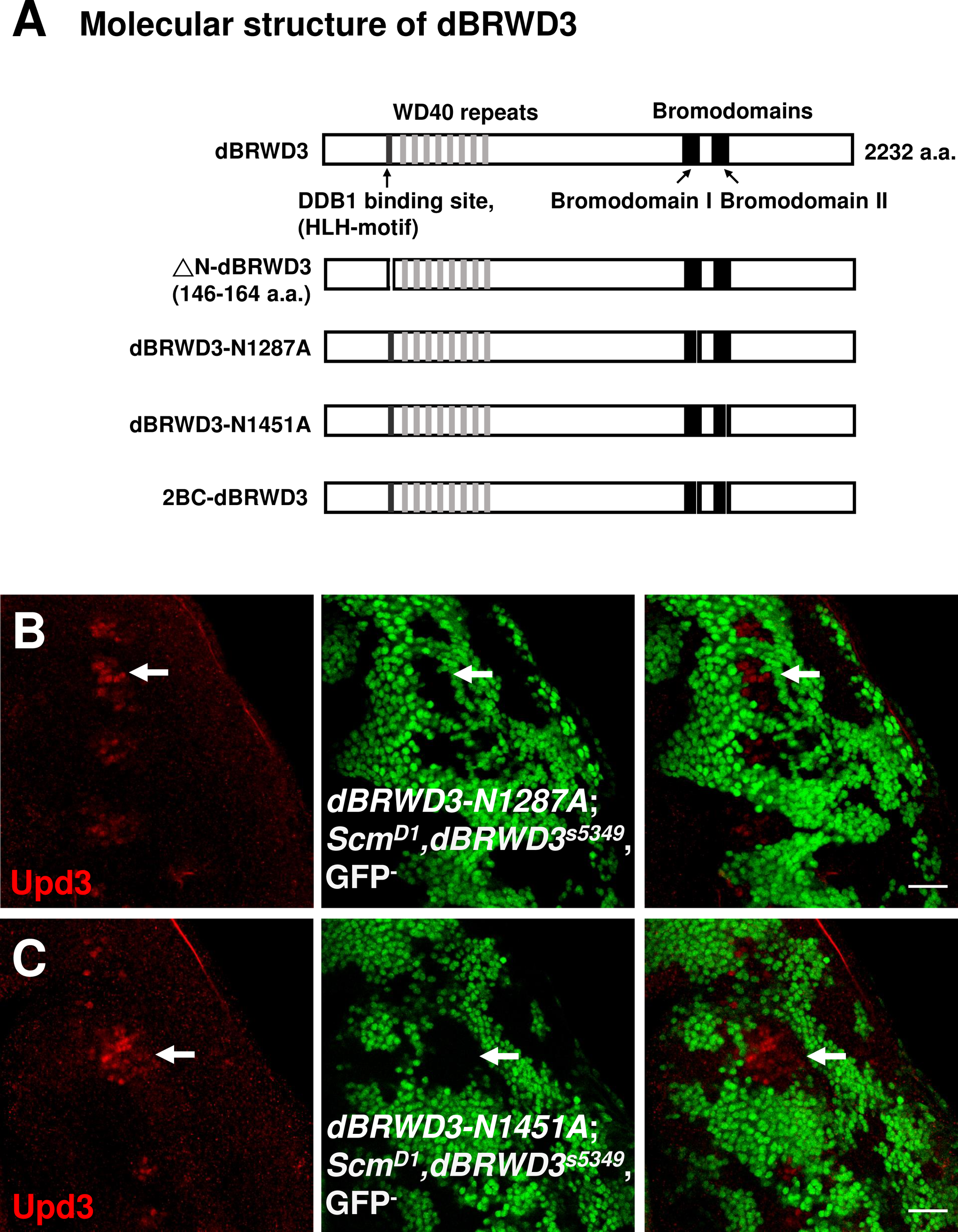

Supplement: S6 Fig — (A) A diagram illustrating the molecular structure of dBRWD3. (B and C) upd3 levels (arrows) in ScmD1, dBRWD3s5349 double-mutant clones complemented with ubiquitously expressed dBRWD3-N1287A-RFP (B) or dBRWD3-N1451A-RFP (C). Scale bars indicate 50μm. (TIF) [file pgen.1006262.s007.tif]

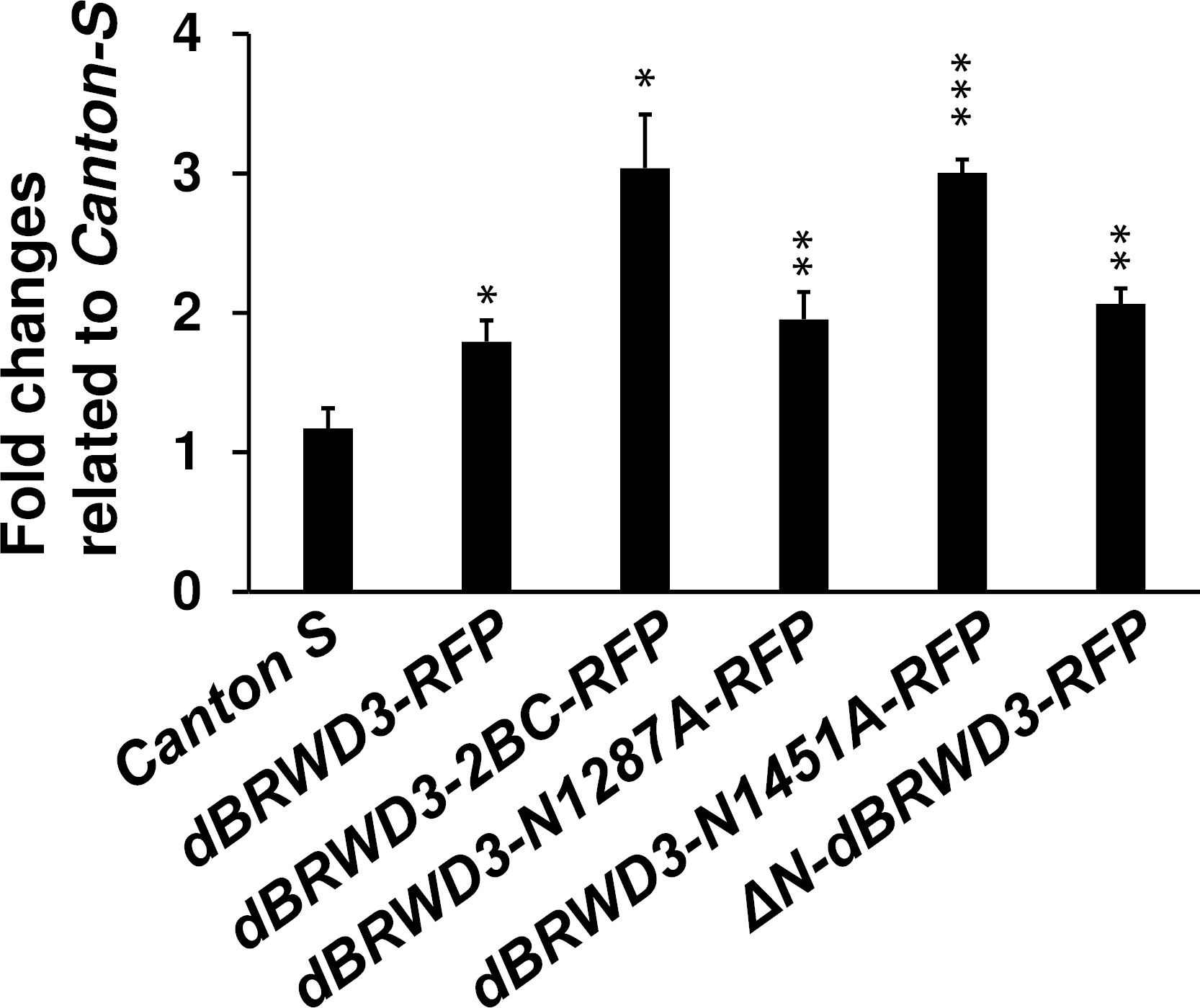

Supplement: S7 Fig — Data represent the mRNA levels of indicated dBRWD3 transgenes plus endogenous dBRWD3 and are shown in means ± S.D., n = 4. *, **, *** indicate P< 0.01, 0.001, 0.0001 respectively in comparison to that in Canton-S by student's t-test. a.u. indicates arbitrary unit. (TIF) [file pgen.1006262.s008.tif]

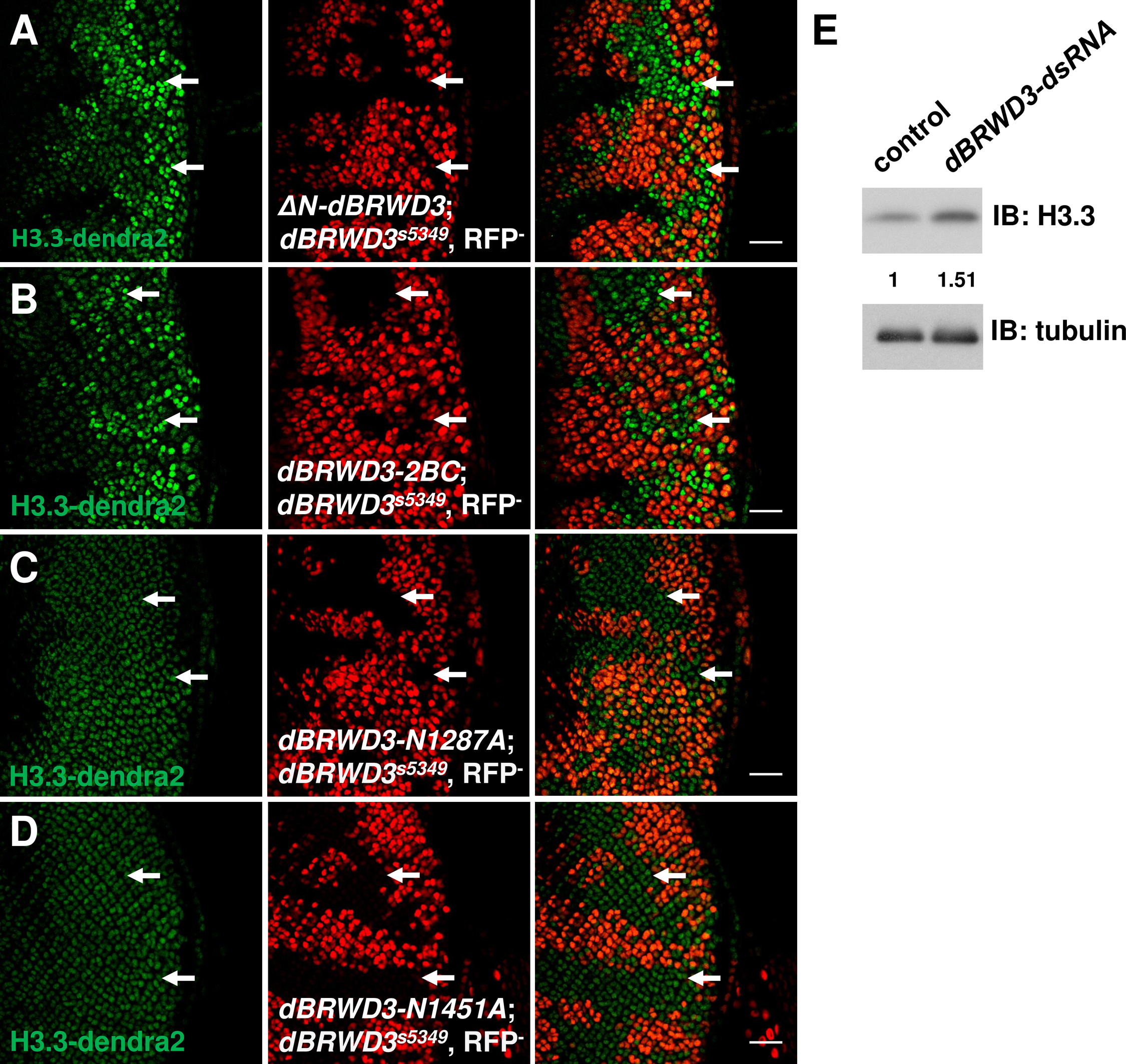

Supplement: S8 Fig — (A-D) H3.3-dendra2 driven by a ubi-promoter was expressed in dBRWD3s5349 mosaic mutant eye discs. dBRWD3s5349 mutant clones were marked by the absence of RFP (arrows), with concomitant expression of ΔN-dBRWD3-RFP (A), dBRWD3-2BC-RFP (B), dBRWD3-N1287A-RFP (C), and dBRWD3-N1451A-RFP (D). Scale bars indicate 50μm. (E) The levels of endogenous H3.3 in dBRWD3-depleted brains and ventral nerve cords. (TIF) [file pgen.1006262.s009.tif]

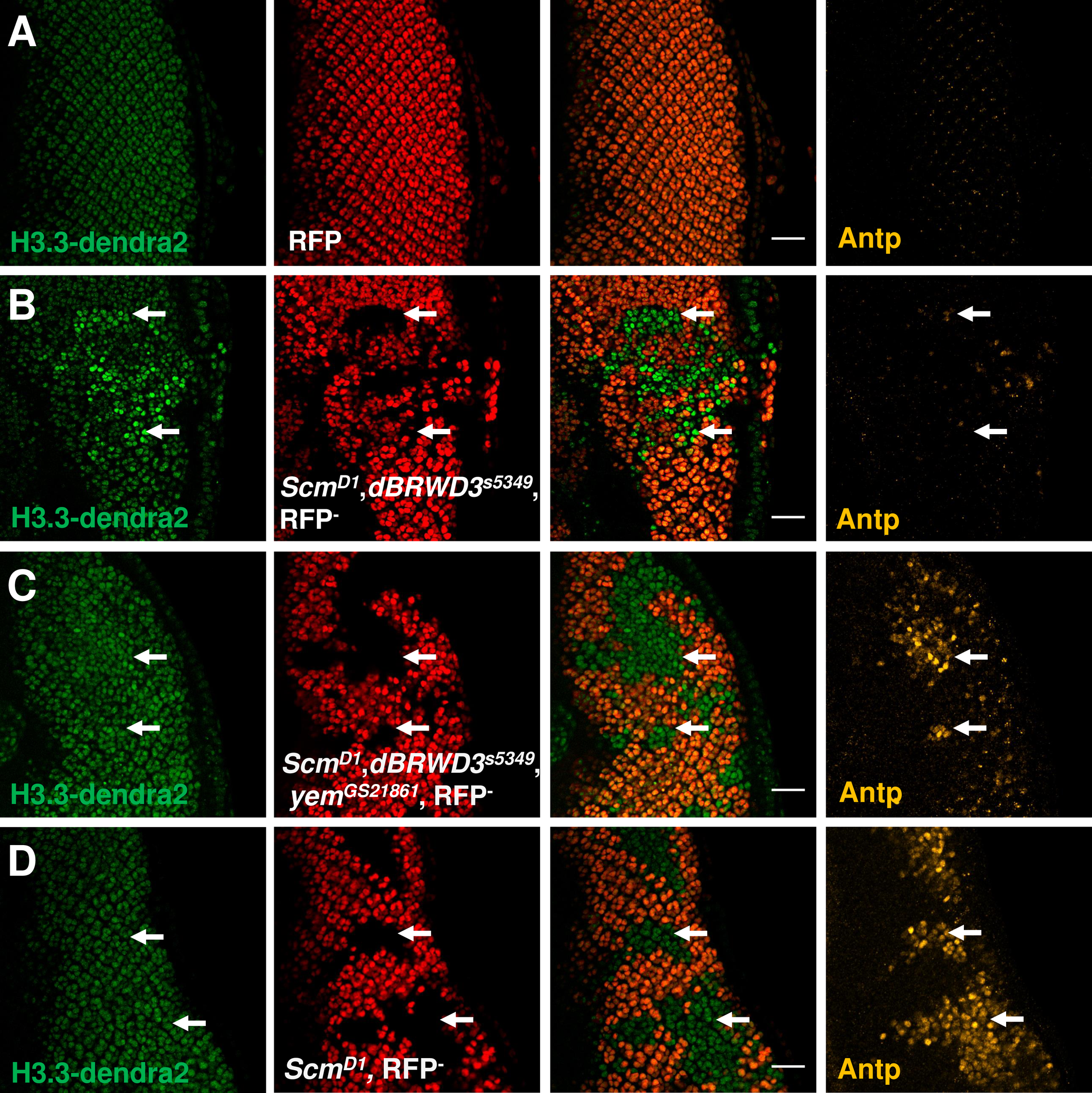

Supplement: S9 Fig — (A) H3.3-dendra2 and Antp levels in wild type eye discs. (B-D) H3.3-dendra2 and Antp levels in ScmD1, dBRWD3s5349, double-mutant clones (B), ScmD1, dBRWD3s5349, yemGS21861 triple-mutant clones (C), and ScmD1 single-mutant clones (D) that were generated in the eye disc and marked by the absence of RFP (arrows). Scale bars indicate 50μm. (TIF) [file pgen.1006262.s010.tif]

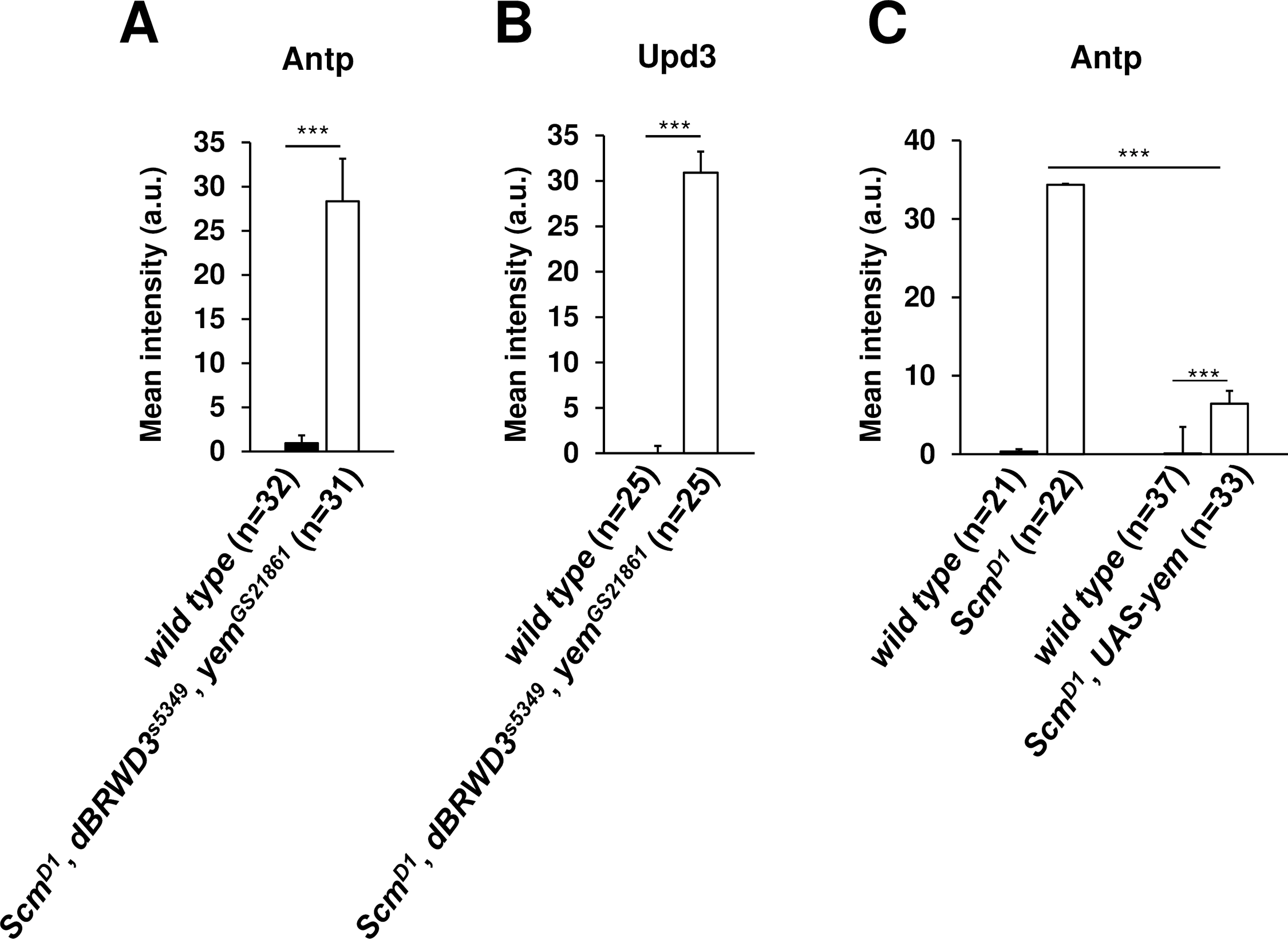

Supplement: S10 Fig — (A and B) The mean intensity of Antp (A) and Upd3 (B) staining in wild type and ScmD1 dBRWD3s5349, yemGS21861 triple-mutant clones. *** indicates p<0.0001 by Student's t-test. (C) The mean intensity of Antp staining in ScmD1 and ScmD1 UAS-yem. *** indicates p<0.0001 by Student's t-test. (TIF) [file pgen.1006262.s011.tif]

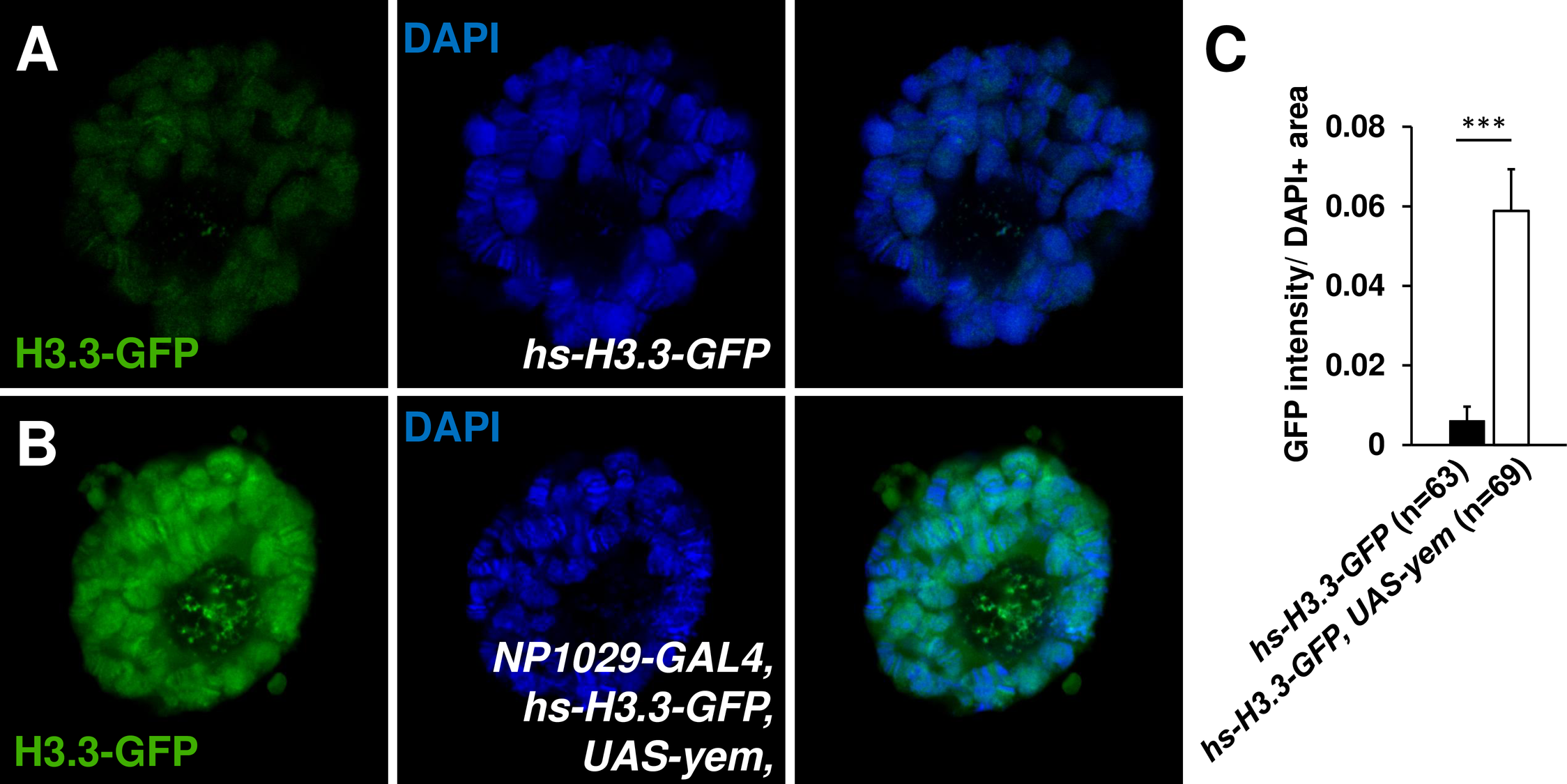

Supplement: S11 Fig — (A and B) Heat-shock inducible H3.3-GFP was expressed in wild-type (A) and yem-expressing (B) salivary glands. (C) The quantification of H3.3-GFP levels in (A) and (B). (TIF) [file pgen.1006262.s012.tif]

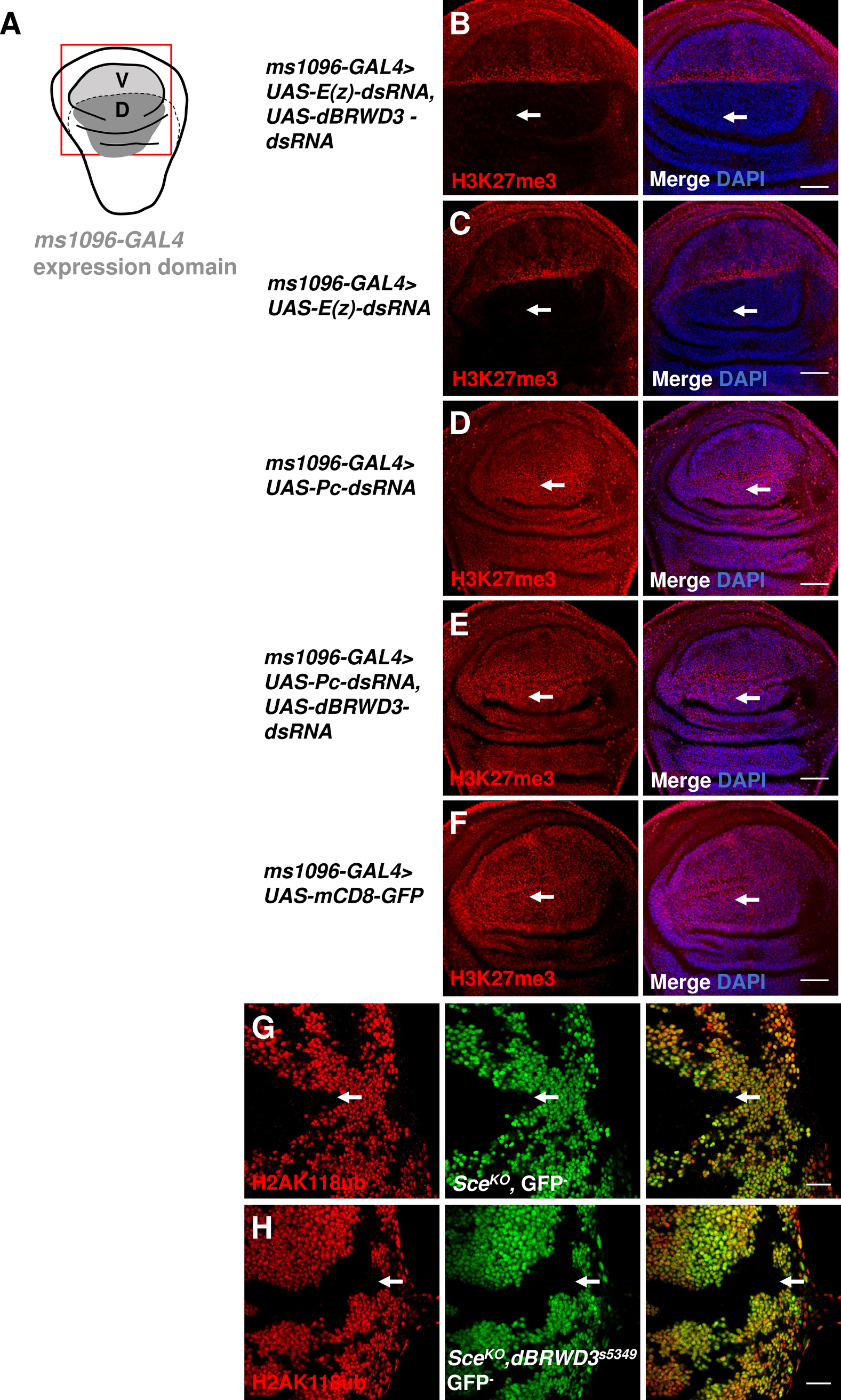

Supplement: S12 Fig — (A) A schematic illustration of the expression domain of ms1096-GAL4. Expression in the dorsal compartment (dark grey) is higher than in the ventral compartment (light grey). (B-F) Immunofluorescence studies of H3K27me3 levels in the E(z), dBRWD3 doubly depleted (B), E(z) depleted (C), Pc, dBRWD3 doubly depleted (D), Pc depleted (E), and control (F) wing discs. (G and H) H2AK118ub levels in SceKO (G) and dBRWD3s5349, SceKO (H) mutant clones. Scale bars indicate 50μm. (TIF) [file pgen.1006262.s013.tif]

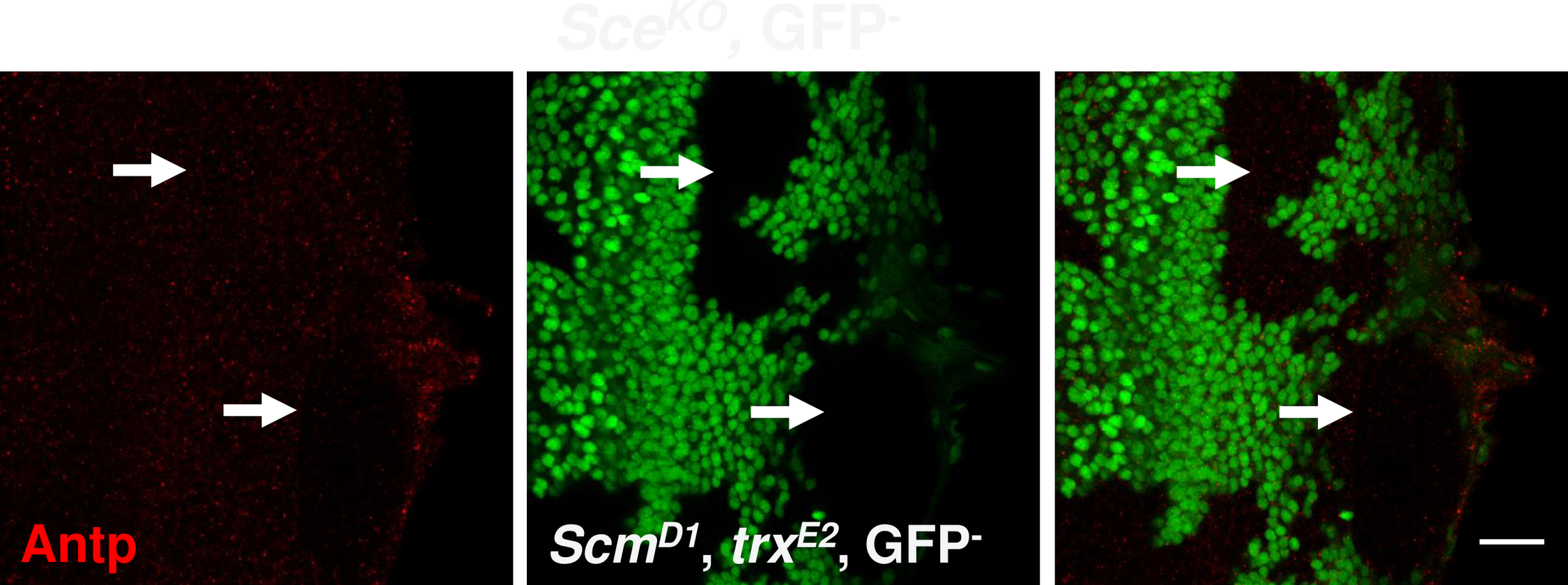

Supplement: S13 Fig — The ectopic expression of Antp in ScmD1, trxE2 double-mutant clones marked by the absence of GFP (arrows). Scale bars indicate 50μm. (TIF) [file pgen.1006262.s014.tif]

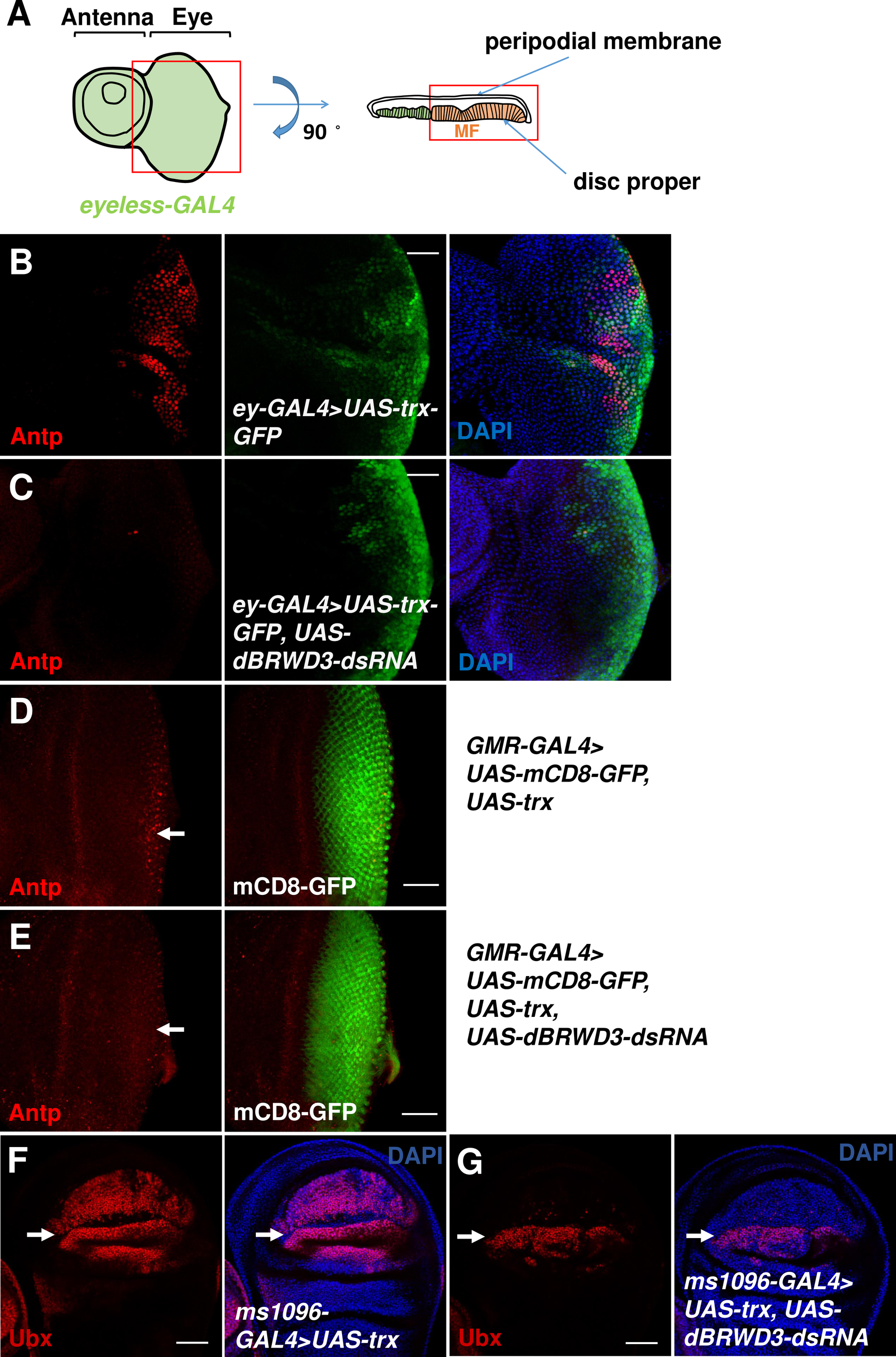

Supplement: S14 Fig — (A) A schematic illustration of the disc proper and the peripodial epithelium of the eye disc. (B and C) Antp levels in the trx-expressing (B) and trx-expressing, dBRWD3-depleted (C) peripodial epithelia. Scale bars indicate 50μm. (D-E) trx was overexpressed under the control of GMR-GAL4. The TRX-induced Antp expression (arrows) in wild-type (D), dBRWD3 depletion (E) backgrounds. (F-G) trx was overexpressed under the control of ms-1096-GAL4. The TRX-induced Ubx expression (arrows) in wild-type (F), dBRWD3 depletion (G) backgrounds. Scale bars indicate 20μm. (TIF) [file pgen.1006262.s015.tif]

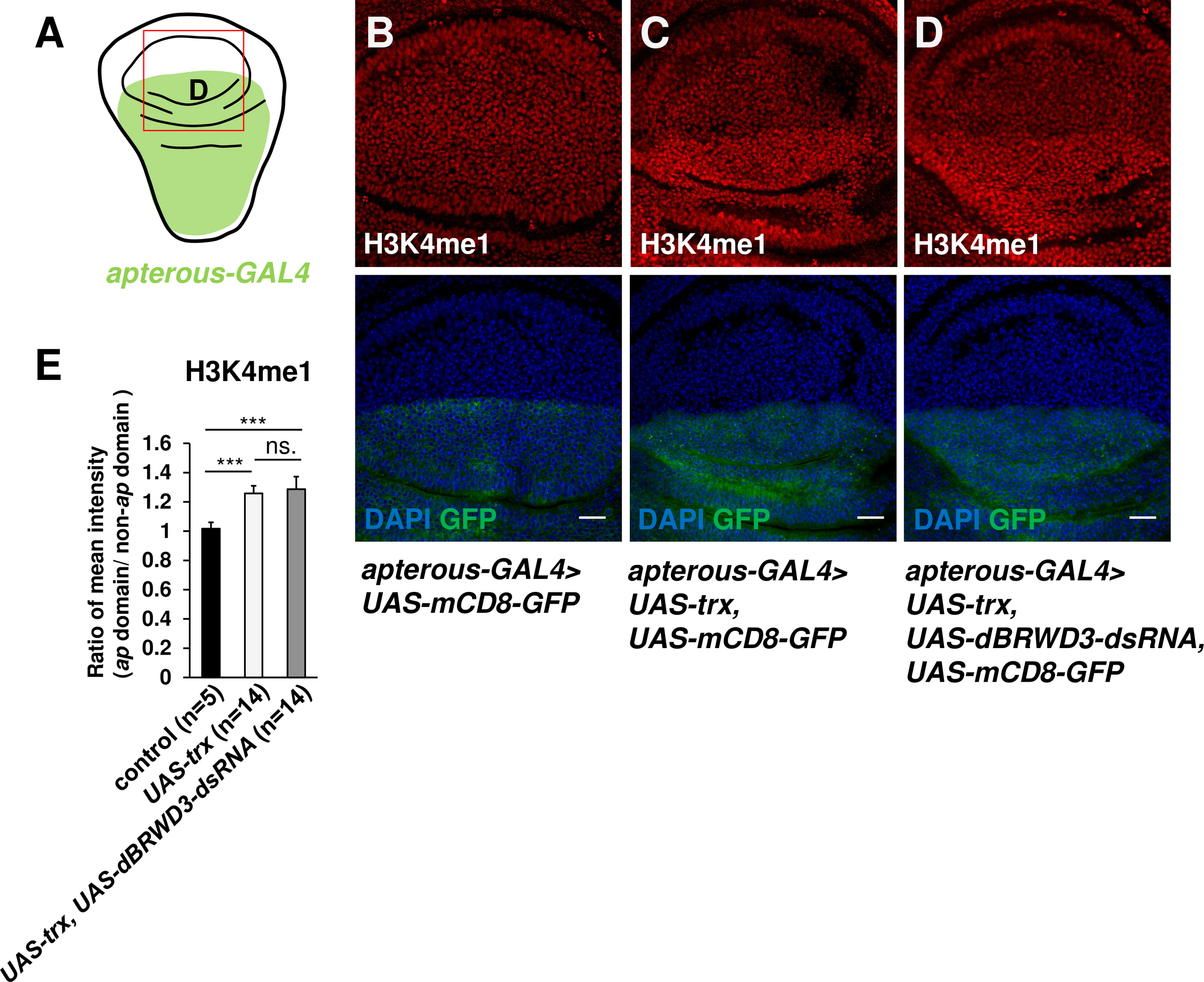

Supplement: S15 Fig — (A) A schematic illustration of the apterous-GAL4 expression region in the 3rd wing imaginal discs. D indicates the dorsal compartment. (B-D) Immunofluorescence studies of H3K4me1 levels in the dorsal compartment of the apterous-GAL4 control (B), trx-expressing (C), and trx-expressing, dBRWD3 depleted (D) wing discs marked by GFP. Scale bars indicate 50μm. (E) A quantitative analysis of H3K4me1 levels in control, trx over-expression, and trx over-expression, dBRWD3-depleted wings. *** indicates p<0.0001 by Student's t-test. ns. indicates not significant. (TIF) [file pgen.1006262.s016.tif]

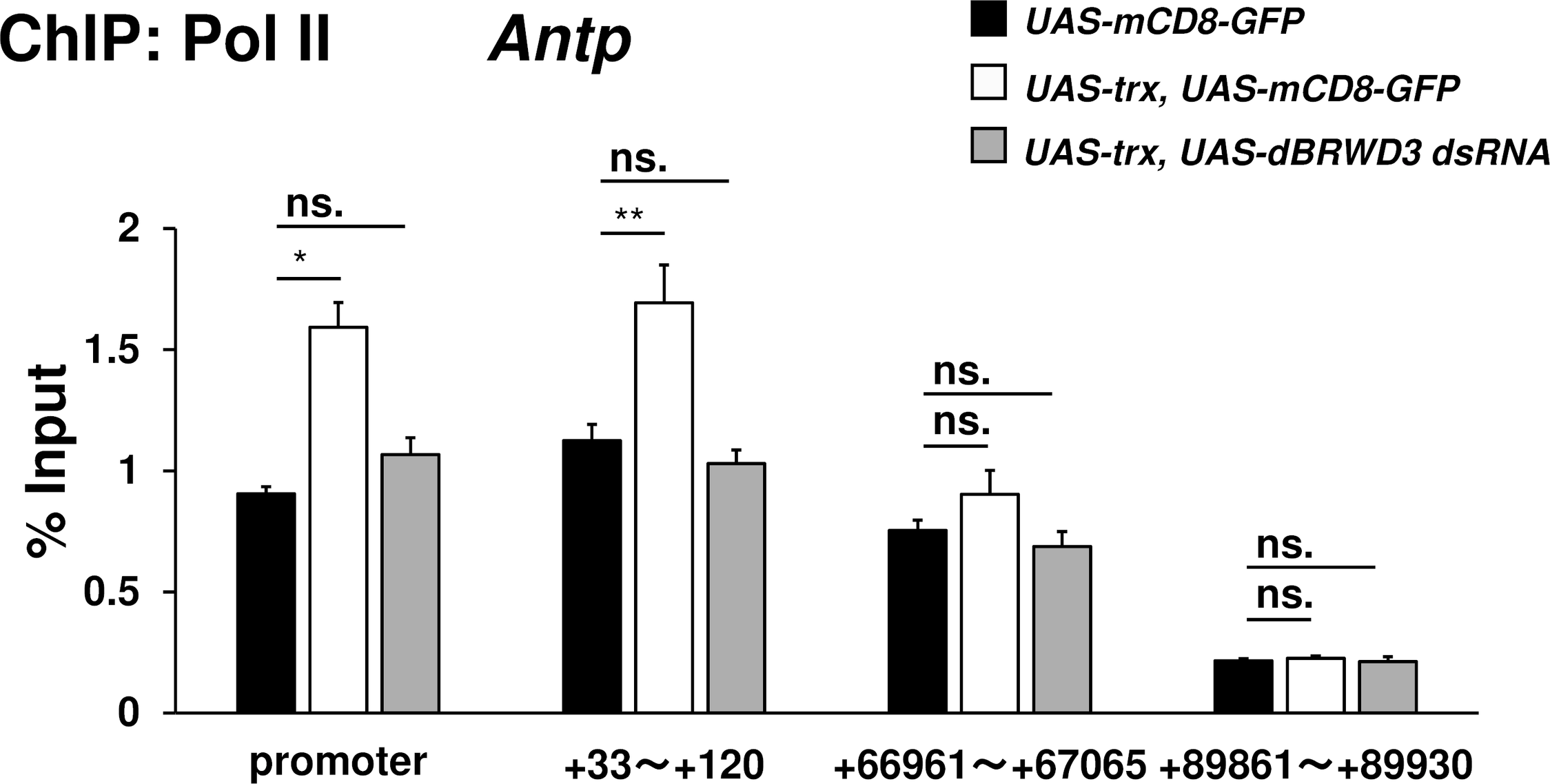

Supplement: S16 Fig — A ChIP-qPCR analysis of PolII levels at the promoters and transcription start sites of Antp in the UAS-mCD8-GFP control, in wings over-expressing trx, or in wings with concurrent trx over-expression and dBRWD3 depletion. ns. indicates not significant. ChIP-qPCR Data are shown as means ± S.D from 4 technical replicates. *, ** indicate P<0.05, 0.01 respectively by Student's t-test. (TIF) [file pgen.1006262.s017.tif]

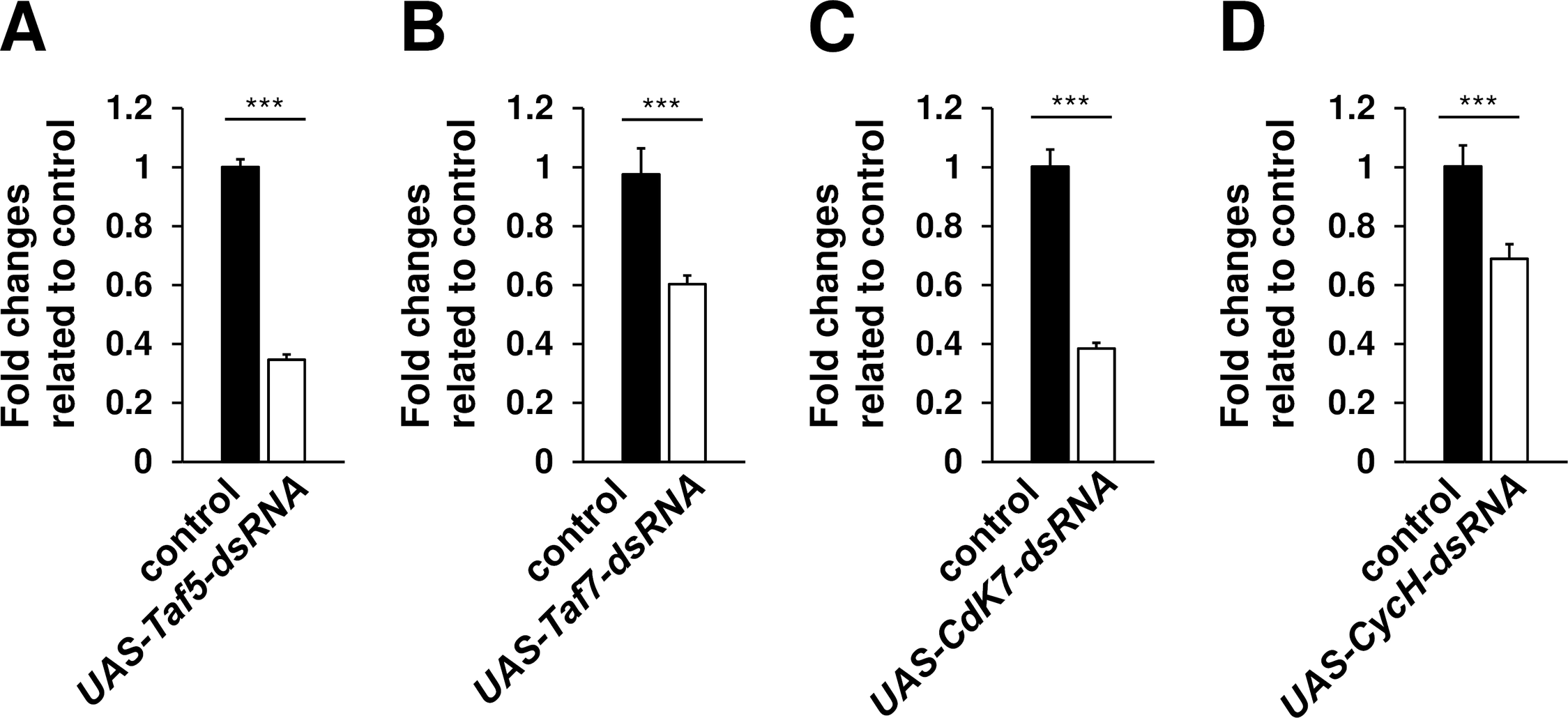

Supplement: S17 Fig — (A-D) A quantitative analysis of the knockdown efficiencies of Taf5 (A), Taf7 (B), Cdk7 (C), and CycH (D) RNAi. *** indicates p<0.0001 by Student's t-test. (TIF) [file pgen.1006262.s018.tif]

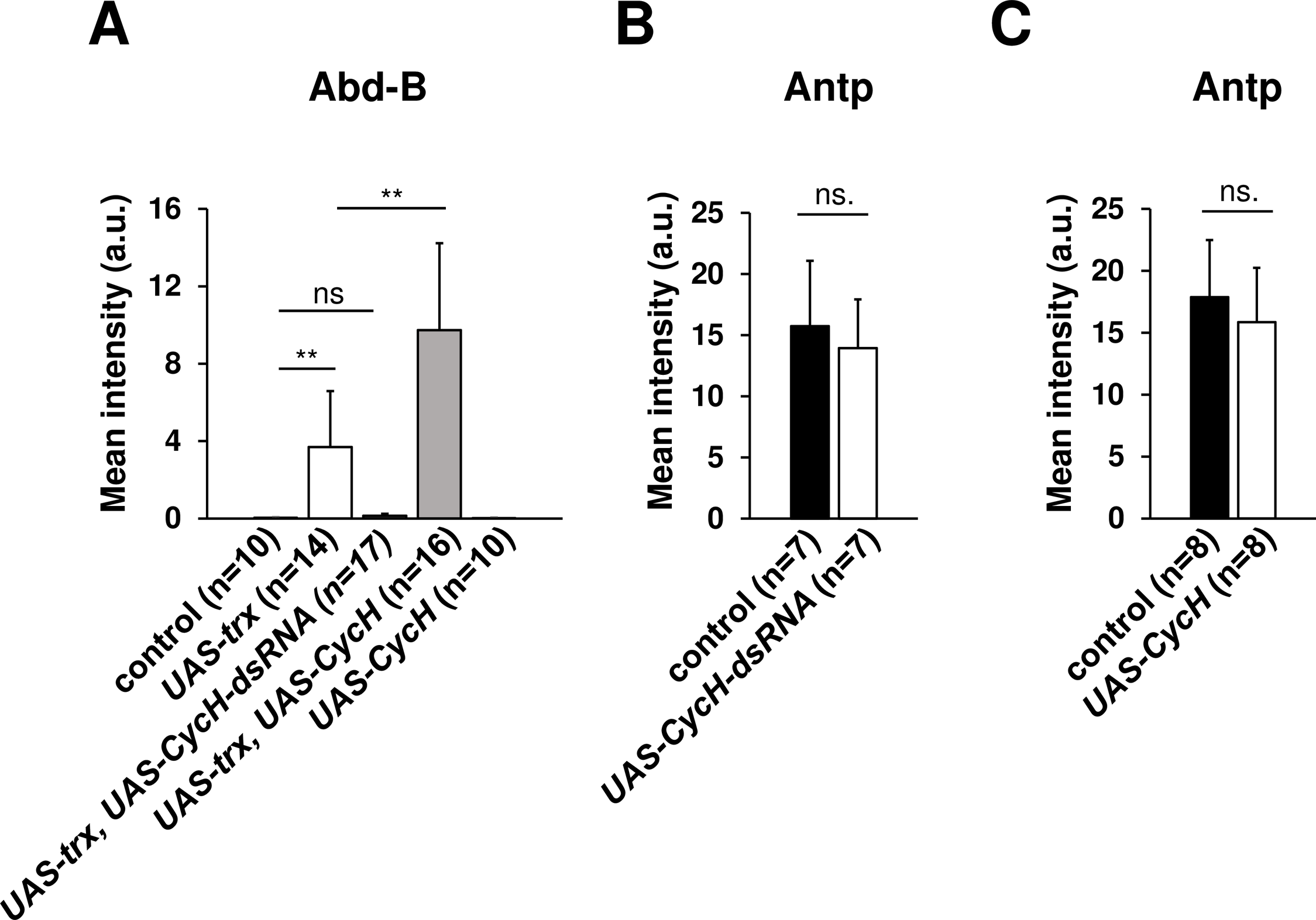

Supplement: S18 Fig — (A) A quantitative analysis of Abd-B expression in the control, Trx-over-expressing, Trx-over-expressing plus CycH knockdown, Trx and CycH-over-expressing, and CycH-over-expressing wings. (B and C) quantitative analyses of orthotopic expression of Antp in the CycH knockdown (B) and the CycH-over-expressing (C) wings. *** indicates p<0.0001 by Student's t-test. ns. indicates not significant. (TIF) [file pgen.1006262.s019.tif]
